# Simultaneous blockade of VEGF-B and IL-17A ameliorated diabetic kidney disease by reducing ectopic lipid deposition and alleviating inflammation response

**DOI:** 10.1038/s41420-023-01304-5

**Published:** 2023-01-16

**Authors:** Zhonglian Cao, Hui Zhao, Jiajun Fan, Yilan Shen, Lei Han, Guangjun Jing, Xian Zeng, Xin Jin, Zeguo Zhu, Qi Bian, Yanyang Nan, Xiaozhi Hu, Xiaobin Mei, Dianwen Ju, Ping Yang

**Affiliations:** 1 Department of Biological Medicines & Shanghai Engineering Research Center of Immunotherapeutic, Fudan University School of Pharmacy, 201203 Shanghai, China; 2 Instrumental Analysis Center, Fudan University School of Pharmacy, 201203 Shanghai, China; 3 Department of Nephrology, Changhai Hospital, Second Military Medical University, 200433 Shanghai, China; 4 Department of Nephrology, Gongli Hospital of Shanghai Pudong New Area, 200135 Shanghai, China

**Keywords:** Drug regulation, Diabetes complications

## Abstract

The pathogenesis of diabetic kidney disease (DKD) is complicated. Current clinical treatments fail to achieve satisfactory efficacy in the prevention of DKD progression, it urgently needs novel and effective treatment for DKD. In this study, we firstly demonstrated that renal lipid metabolism abnormality and inflammation significantly changed in DKD conditions by mining public transcriptomic data of DKD patient samples. KEGG analysis further exhibited the critical role of vascular endothelial growth factor B (VEGF-B) and interleukin 17A (IL-17A) signal pathways in DKD progression, indicating that VEGF-B and IL-17A might be the promising targets for DKD treatment. Then the potential of a novel combination therapy, anti-VEGF-B plus anti-IL-17A antibody, was evaluated for DKD treatment. Our results demonstrated that simultaneous blockade of VEGF-B and IL-17A signaling with their neutralizing antibodies alleviated renal damage and ameliorated renal function. The therapeutic effectiveness was not only related to the reduced lipid deposition especially the neutral lipids in kidney but also associated with the decreased inflammation response. Moreover, the therapy alleviated renal fibrosis by reducing collagen deposition and the expression of fibronectin and α-SMA in kidney tissues. RNA-seq analysis indicated that differential expression genes (DEGs) in db/db mice were significantly clustered into lipid metabolism, inflammation, fibrosis and DKD pathology-related pathways, and 181 of those DEGs were significantly reversed by the combinatory treatment, suggesting the underlying mechanism of administration of anti-VEGF-B and anti-IL-17A antibodies in DKD treatment. Taken together, this study identified that renal lipid metabolism abnormality and inflammation were critically involved in the progression of DKD, and simultaneous blockade of VEGF-B and IL-17A signaling represents a potential DKD therapeutic strategy.

## Introduction

Diabetic kidney disease (DKD) is one of the most severe complications of diabetes. The disease is mainly characterized by glomerular hypertrophy, thicken basement membrane, glomerular sclerosis, podocyte loss, mesangial matrix hyperplasia as well as expansion, and progressive renal fibrosis [1, 2]. They gradually lead to proteinuria, a decrease in glomerular filtration rate (GFR), and end-stage renal disease (ESRD) in patients [3, 4]. Due to the limited understanding of the pathogenesis of DKD, current clinical treatments mainly focus on glucose and blood pressure control and lifestyle changes, but they exerted limited efficacy in ameliorating the progression of DKD [5–7]. Thus, trying to explore DKD pathogenesis and accelerate the development of innovative and efficacious therapeutic approaches is urgently needed before kidney damage becomes irreversible.

Recent evidence has indicated that renal ectopic lipid accumulation is correlated with the progression of renal diseases, especially DKD. The excess lipid droplets lead to renal damage [8]. Vascular endothelial growth factor B (VEGF-B), a novel member of the VEGFs family, functions as the lipid transport [9]. It is abundantly expressed in vigorous metabolic tissues such as muscles, kidneys and the heart. It triggers signals to the endothelium by interaction with VEGF receptor 1 (VEGFR-1) and the co-receptor neuropilin1 (NRP-1), then leads to transcriptional upregulation of the fatty acid transport protein 3,4 (FATP3, FATP4) which can transport the circulating fatty acids into peripheral organs such as skeletal muscle, heart, and kidney [10]. In db/db mice, genetic deletion of VEGF-B inhibits ectopic lipid accumulation and prevents the progression of DKD [5]. These findings suggest that blocking VEGF-B signaling may be a promising approach for DKD treatment.

Although DKD is mainly considered as a metabolic disease, growing evidence suggests that proinflammatory cytokines extensively participate in the progression of DKD [11–15]. Among them, Interleukin 17A (IL-17A) has been well-documented for its role of a cytokine in driving autoimmune and inflammatory disease [16], it may also promote the inflammatory injury of the kidney [17]. The other report indicates that IL-17A is involved in acute and chronic kidney disease [18], the renal injury is ameliorated in IL-17A deficient DKD mice compared with wild-type mice [19].

The lipid metabolism abnormality can lead to ectopic fat distribution in the peripheral organs including the kidney, it may be one of the accelerators of inflammation [20], and the inflammation response can, in turn, promote renal injury and dysfunction [14]. All these hints that lipid metabolism abnormality, ectopic fat accumulation and inflammation may play critical roles in the processing of DKD.

In the current study, we, therefore, analyzed transcriptomics profiles of kidney samples from 112 patients and 50 normal controls from the GEO database [21]. It was identified that the renal lipid metabolism abnormality and inflammation were significantly changed in the DKD condition. Further analysis indicated that lipid transport regulating factor VEGF-B and the pro-inflammatory cytokine IL-17A were closely involved in DKD progression, indicating them to be the potential targets for DKD treatment. Thus, the therapeutic effect of simultaneously blocking VEGF-B and IL-17A signaling pathways with their neutralizing antibodies was evaluated for the first time in leptin-receptor-deficient db/db mice, a robust and progressive DKD animal model. We found that eliminating both VEGF-B and IL-17A significantly ameliorated renal dysfunction and disease progression in DKD mice by reducing the ectopic lipid deposition, and inflammation and inhibiting the renal fibrosis response. For the mechanistic study, the transcriptomic analysis was performed, and the data showed that the differential expression gene (DEGs) were mainly enriched in lipid metabolism, IL-17A-dependent inflammation, fibrosis and DKD pathology-related pathways. Importantly, the combined administration of anti-IL-17A and anti-VEGF-B antibodies remarkably reversed the genes which are closely related to lipid metabolism, inflammation, and renal fibrosis. Our study indicated that both VEGF-B and IL-17A signal pathways played critical roles in DKD and simultaneously targeting them represented a novel efficacious therapeutic approach to treat DKD.

## Results

### The renal lipid metabolism abnormality and inflammation were involved in the progress of DKD patients

Our previous study revealed that dosing recombinant cytokine IL-22 for enhancing the repairment of renal damage showed effective synergy with VEGF-B antibody (for rebalancing lipid metabolism) to ameliorate DKD progression [22], and transcriptomics analysis indicated that IL-17A signaling was highly involved in anti-VEGF-B-IL-22 treatment [22]. Other evidence also highlighted the critical roles of IL-17A signaling in renal damage and its potential clinical value as a therapeutic target for DKD treatment [17, 18, 23]. We, therefore, hypothesize that the IL-17A signaling pathway is important for the effectiveness of VEGF-B-based DKD therapy. Integrated analysis of massive published genomics data has contributed to many novel findings and is widely used to test hypothesis [24]. We, therefore, constructed a human DKD gene expression dataset including 50 normal controls and 112 DKD samples for integrated analysis (Supplementary Table 1, Supplementary Fig. 1).

**Fig. 1:**
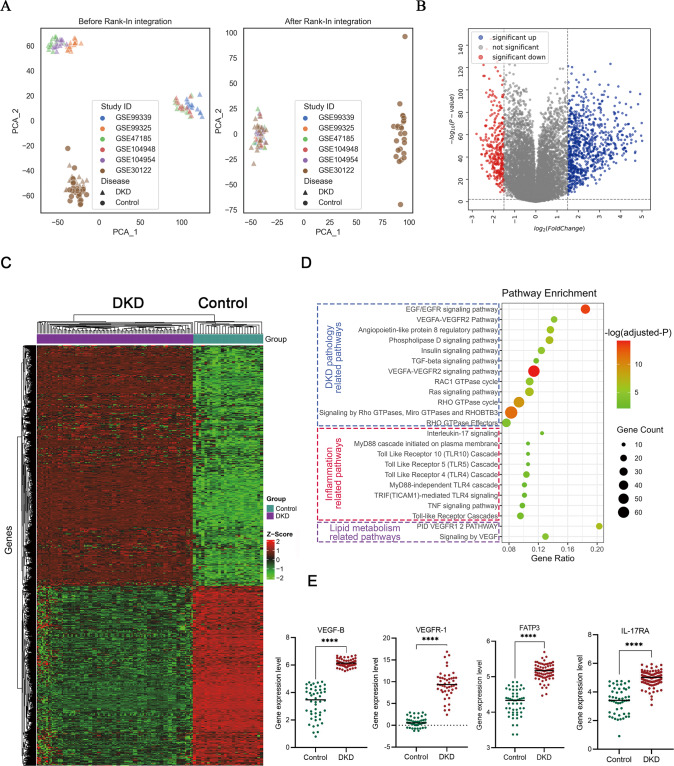
Bioinformatics analysis of publicly available gene expression dataset of DKD patients. **A** Distribution of samples of control and DKD patients before and after Rank-In integration. The source study IDs (in GSExxx format) were indicated by different colors. **B** Differential expressed genes (DEGs) analysis using the limma algorithm. log2 (fold change) of 1.5 and log10 (FDR-adjusted *P* values) of 0.01 were indicated with dash lines in the plot. **C** DEGs were represented in the heatmap by hierarchical clustering of both genes and samples according to their gene expression *Z*-scores. **D** Significantly enriched signaling pathways in three categories were visualized in the bubble chart. **E** Gene expression values of VEGF-B, VEGFR-1, FATP3 and IL-17RA between control and DKD samples were compared with Student’s *t*-test, *n* = 50-112. ^****^P < 0.0001.

As expected, samples from different source studies showed obvious batch effect and the Rank-In algorithm effectively normalized samples by removing the batch effect but keeping biological variations (Fig. 1A). The subsequent DEGs analysis indicated that 463 genes and 83 genes were significantly upregulated and downregulated in DKD samples, respectively (Fig. 1B, C). To assess the functional meanings of these DEGs at the system level, we performed pathway enrichment analysis using Metascape webserver. As shown in Fig. 1D, 463 upregulated DEGs are mainly overrepresented in DKD pathology-related pathways (e.g., EFG/EGFR pathway, Rho GTPases signaling, insulin signaling pathway), inflammation pathways (e.g., IL-17 signaling, TNF signaling pathway, toll-like receptor signaling), and lipid metabolism-related pathways (VEGF signaling) (full pathway list was provided in Supplementary Table 2). Interestingly, among inflammation-related pathways, IL-17A signaling was significantly enriched with the highest gene ratio (the ratio of enriched genes in all genes of the pathway). We further extracted the gene expression levels of VEGF-B, VEGFR-1, FATP3 and IL-17RA, key mediators of VEGF-B signaling and IL-17 signaling, and found that their gene expression productions were significantly higher in DKD samples than in normal control (Fig. 1E). Together with our previous study, these bioinformatics analysis results demonstrated that both VEGF-B and IL-17A signaling were highly involved in DKD, suggesting the potential therapeutic value of simultaneously inhibiting those two pathways in DKD treatment.

### Neutralizing of VEGF-B and IL-17A simultaneously markedly ameliorated renal function and alleviated renal injury in DKD mice

To evaluate the therapeutic efficacy of simultaneously blocking VEGF-B and IL-17A signaling, their neutralizing antibodies anti-VEGF-B and anti-IL-17A were employed to explore their roles in improving renal function and alleviating renal damage in the DKD mouse model which is widely used [25].

Firstly, anti-VEGF-B and anti-IL-17A were expressed and purified, they both had high purity which was characterized by high-performance size-exclusion chromatography (Supplementary Fig. 2A, B). Anti-VEGF-B could effectively block the interaction of VEGF-B and its receptor VEGFR-1 in HUVEC cells, and anti-IL-17A could inhibit the IL-6 production by blocking the IL-17A signaling in NIH-3T3 cells, the results showed that anti-VEGF-B and anti-IL-17A would efficiently antagonize VEGF-B and IL-17A, respectively (Supplementary Fig. 2C, D). The surface plasmon resonance analysis was used to evaluate the affinity, the results demonstrated that anti-VEGF-B and anti-IL-17A had excellent affinities to VEGF-B and IL-17A at 0.188 and 0.0371 nM, respectively (Supplementary Fig. 2E, F). The toxicity of anti-VEGF-B and anti-IL-17A in vivo was also evaluated. Histological observation showed that there was no obvious pathological damage in the heart, liver, spleen, lung and kidney after 7 days of anti-VEGF-B and anti-IL-17A administration, indicating that anti-VEGF-B and anti-IL-17A had good biosafety (Supplementary Fig. 3).

**Fig. 2:**
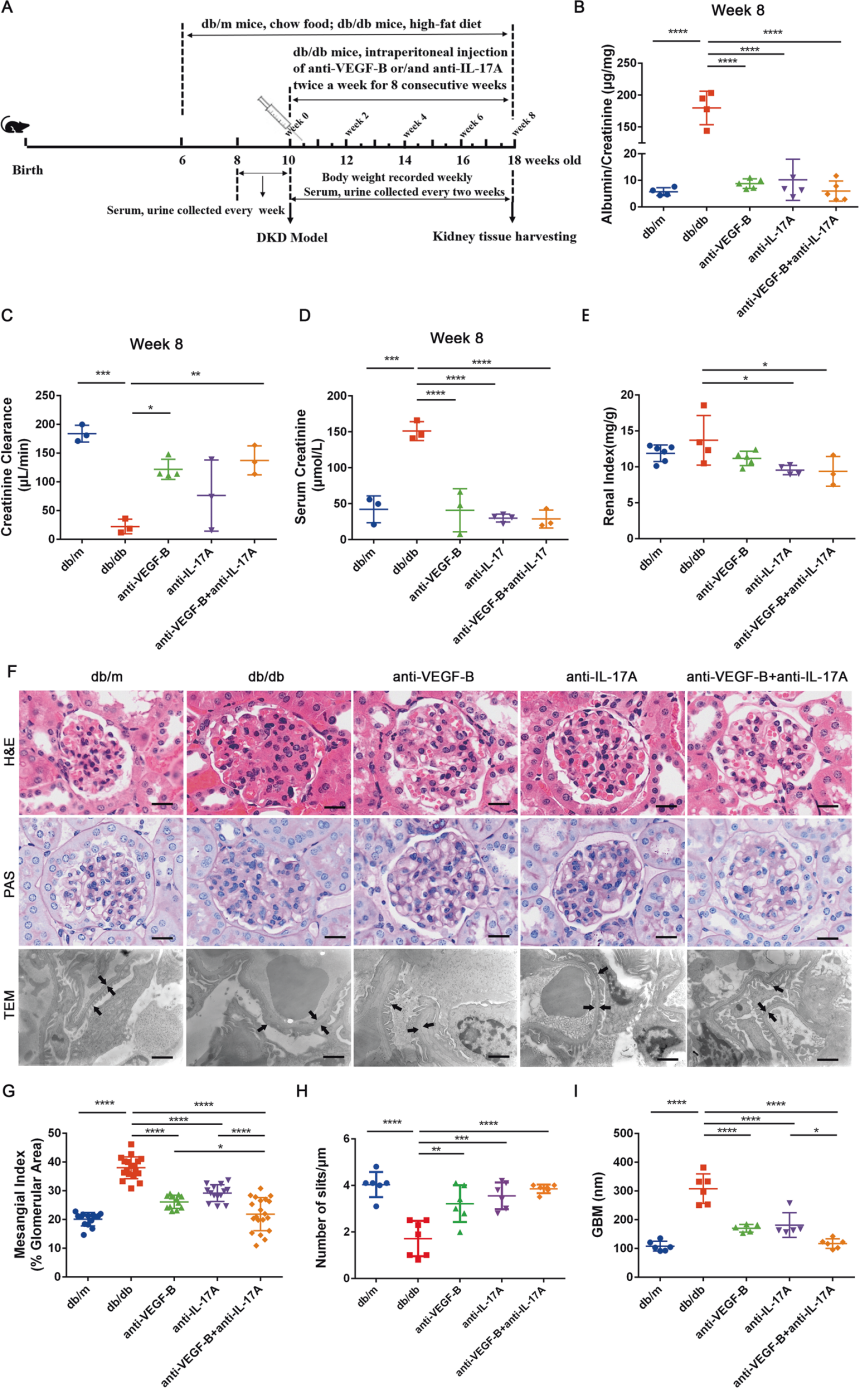
Neutralizing of VEGF-B and IL-17A simultaneously alleviated renal damage and improved renal function in established DKD mice. **A** The treatment scheme of anti-VEGF-B or/and anti-IL-17A for db/db mice. **B** Quantitative analysis of urine ACR at 8th week after treatment. **C** Quantitative analysis of serum Ccr at 8th week after treatment. **D** The content of mice serum creatinine was quantified at 8th week after the administration. **E** Analysis of renal index (renal weight/body weight). **F** Images of kidney sections by HE staining (scale bar:10 μm), periodic acid-Schiff (PAS) staining (scale bar: 10 μm) and TEM (scale bar:1 μm). **G** Glomerular mesangial index was quantified according to PAS staining (*n* = 4 mice in each group, per section/per mice, 5 glomeruli/per section). **H** The number of podocyte slits per micrometer (slit density) was quantified according to TEM images (*n* = 3 mice in each group, per section/per mice, 2 slit density/per section). **I** Quantification of GBM thickness according to TEM images (*n* = 3 mice in each group, per section/per mice, 2 GBM thickness/per section). GBM thickness and podocyte foot processes are indicated with arrowheads. (scale bar: 1 μm). The data were presented as means ± SD, *n* = 3–6 mice in each group (**B–E**), ^****^P < 0.0001, ^***^P < 0.001^, **^P < 0.01, ^*^P < 0.05^.^ The statistical significance was analyzed by one-way ANOVA with Tukey’s multiple-comparisons test.

Secondly, in vivo therapeutic efficacy was evaluated on the DKD mouse model (Fig. 2A). At the beginning, db/db mice were subjected to standard HFD feeding to successfully establish the DKD model which was confirmed by the fact that standard HFD feeding resulted in a remarkable increase in blood glucose and ACR levels (Supplementary Fig. 4). The results indicated that ACR levels in db/db mice were significantly increased compared with db/m mice, and this increase was almost reversed after 8 weeks’ treatment with anti-VEGF-B and anti-IL-17A. Althrough anti-VEGFB or anti-IL-17A alone also significantly decreased the level of ACR, their therapeutic effect was slightly lower than that of the combined group (Fig. 2B, Supplementary Fig. 5A–C). The Ccr level was significantly decreased in db/db mice but was obviously increased in the treated group. More importantly, it showed a better therapeutic effect in the combination treatment than in anti-VEGFB or anti-IL-17A alone treatment after 8 weeks of therapy (Fig. 2C, Supplementary Fig. 5D–F). Compared with the model control, serum creatinine level (Fig. 2D) was significantly decreased in all treated groups and the combination treatment exhibited better effects compared with single treatments. In addition, the renal index did not reduce, while the combination treatment exhibited a significant decrease (Fig. 2E).

Next, H&E staining, PAS staining and TEM analysis were employed to determine the renal pathological alterations, glomerular mesangial matrix expansion, podocyte foot process effacement and glomerular basement membrane thickness of renal tissues (Fig. 2F). The H&E staining result showed that the combined treatment showed less kidney injury than anti-VEGFB or anti-IL-17A alone treatment. The PAS staining illustrated that glomerular mesangial matrix expansion was very serious and mesangial index was significantly higher in db/db mice than that in db/m mice, and it reduced significantly after anti-VEGF-B and anti-IL-17A treatment in db/db mice. More importantly, the therapeutic efficacy of the combination treatment was better than anti-VEGF-B or anti-IL-17A alone (Fig. 2G). The TEM results indicated that the podocyte foot process effacement could be distinctly reversed by comparing the number of slits after the combined therapy of anti-VEGF-B and anti-IL-17A (Fig. 2H), the glomerular basement membrane thickness in db/db mice was also obviously decreased (Fig. 2I).

The body weight of db/db mice was significantly heavier than that of db/m mice, there were no differences among treated groups (Supplementary Fig. 6A). However, we observed fat pads accumulated in the subcutaneous tissue of the abdomen, which might be caused by the fact that lipids were shunted to the white adipose tissue after combination treatment for 8 weeks. The postprandial blood glucose levels of db/db mice were significantly higher than the matched db/m mice, but there was no difference in anti-VEGF-B or anti-IL-17A treated groups. The glucose level decreased significantly in the mice receiving combined therapy (Supplementary Fig. 6B). The IPGTT result illustrated that there was no significant difference in each treated group compared with the db/db mice (Supplementary Fig. 6C).

Taken together, these findings suggested that combined treatment with anti-VEGF-B and anti-IL-17A markedly ameliorated renal function and alleviated renal injury in DKD mice.

### The combined treatment of anti-VEGF-B and anti-IL-17A obviously alleviated renal fibrosis in db/db mice

**Fig. 3:**
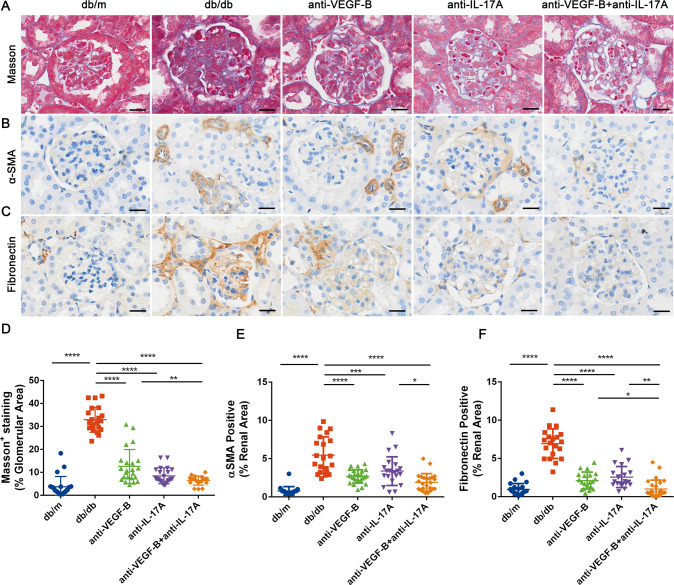
Anti-VEGF-B and anti-IL-17A treatment simultaneously inhibited the renal fibrosis response in *db/db* mice of the DKD model. **A** Deposition of collagen fiber was determined according to Masson staining of *db/db* mice kidney sections (scale bar: 10 μm). **B** and **C** Deposition of Collagen fiber deposition including α-SMA and fibronectin in kidney sections by immunohistochemistry analysis (scale bar: 10 μm). **D** Quantification of the collagen fiber deposition of Masson staining of *db/db* mice kidney sections. **E** and **F** Quantification of the expression of fibrosis markers of fibronectin and α-SMA in kidney sections in DKD mice after anti-VEGF-B or/and anti-IL-17A treatment. The data were presented as means ± SD, *n* = 4 mice in each group, per section/per mice, 5 glomerulus or tubulointerstitial/per section (**D–F**), ^****^P < 0.0001, ^**^P < 0.01, ^*^P < 0.05. The statistical significance was analyzed by one-way ANOVA with Tukey’s multiple-comparisons test.

Renal function is closely associated with renal fibrosis in DKD [26]. Therefore, it was necessary to examine whether simultaneous inhibition of VEGF-B and IL-17A could alleviate renal fibrosis in db/db mice. Masson staining (Fig. 3A), immunohistochemical staining of fibronectin (Fig. 3B) and α-SMA (Fig. 3C) were conducted to determine the efficacy of anti-VEGF-B or/and anti-IL-17A on renal fibrosis. The results of Masson staining showed severe renal collagen deposition in db/db mice, it decreased significantly in anti-VEGF-B or/and anti-IL-17A treated groups. By comparing the percentage of positive Masson staining area in the whole area, the collagen deposition reduced most obviously in the combined therapy group (Fig. 3D). Besides, the results of immunohistochemical staining showed that fibronectin (Fig. 3E) and α-SMA (Fig. 3F) levels in kidney tissues increased significantly in db/db mice compared with the normal mice, the therapy of anti-VEGF-B or/and anti-IL-17A alleviated glomerular and interstitial fibrosis by inhibition the expression of fibronectin and α-SMA, especially the fibronectin expression. Collectively, these results suggested that the combined treatment of anti-VEGF-B and anti-IL-17A alleviated renal fibrosis in db/db mice.

### Treatment of the neutralizing VEGF-B and IL-17A antibodies simultaneously reduced the renal ectopic lipid deposition

**Fig. 4:**
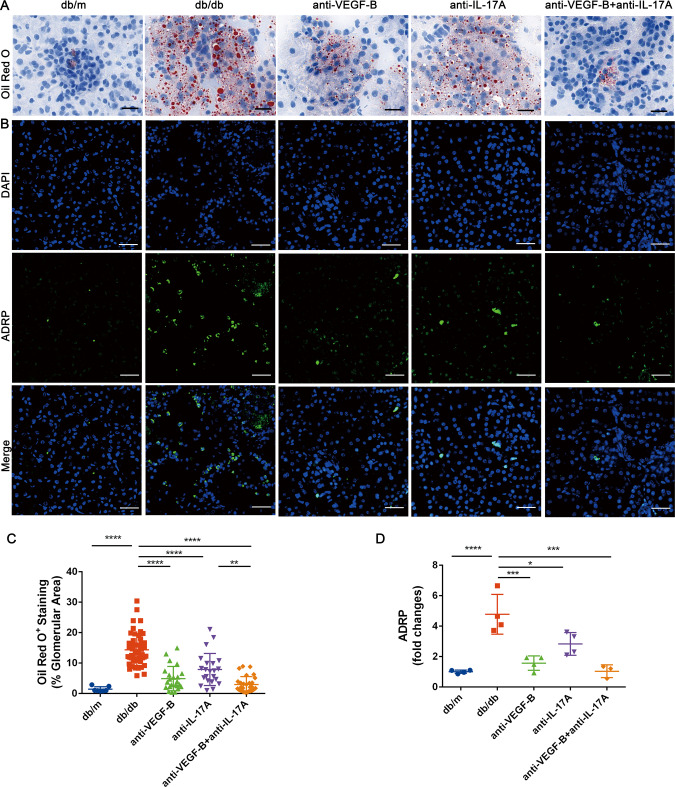
Treatment of the neutralizing VEGF-B and IL-17A antibodies simultaneously reduced the renal ectopic lipid accumulation. **A** Oil Red O staining was used to assess the neutral lipids (NLs) in kidney sections (scale bar: 10 μm). **B** ADRP expression was used to assess the lipid droplets (LDs) by immunofluorescence analysis (scale bar: 10 μm). **C** Quantitative analysis of Oil Red O staining of kidney sections, *n* = 4 mice in each group, per section/per mice, 2–5 glomerulus or tubulointerstitial/per section. **D** Quantitative analysis of the fluorescent intensity of ADRP, *n* = 3–4 mice in each group, per section/per mice, per positive area/per section. The data were presented as means ± SD, ^****^P < 0.0001, ^***^P < 0.0001, ^**^P < 0.01, ^*^P < 0.05^.^ The statistical significance was analyzed by one-way ANOVA with Tukey’s multiple-comparisons test.

It demonstrated that ectopic lipid deposition in renal was highly involved in the progression of DKD. It has been reported that lipids accumulated in the form of lipid droplets [27], excessive lipid droplets were associated with renal dysfunction in DKD patients and experimental models [28]. Targeting VEGF-B-mediated renal lipid accumulation obviously improved the renal function in Vegfb-ablated HFD-fed mice [5]. We therefore investigated if simultaneous blockade of VEGF-B and IL-17A signaling could reduce renal ectopic lipid accumulation. Oil red O staining was used to assess the neutral lipids (NLs) (Fig. 4A), the ADRP expression was used to assess the lipid droplets (LDs) (Fig. 4B). The results indicated that the lipids especially the neutral lipids increased significantly in renal tissue of db/db mice, and they reduced significantly not only in anti-VEGF-B or anti-IL-17A treated group but also in the combined treatment group. Especially the combination treatment was more effective than the anti-IL-17A treatment (Fig. 4C). The expression of ADRP increased in db/db mice and it was effectively decreased by anti-VEGF-B or/and anti-IL-17A therapy, it indicated that accumulation of LDs in kidney reduced after the administration, especially the combination treatment was more efficient than the anti-IL-17A alone treatment (Fig. 4D). Serum triglyceride and free fatty acid were then investigated, the results demonstrated that they significantly increased in db/db mice compared with the db/m mice, the triglyceride significantly reduced in each group (Supplementary Fig. 7A), the free fatty acid decreased obviously in anti-VEGF-B and combination-treated groups but not in anti-IL-17A treated group (Supplementary Fig. 7B).

### Inhibition of NF-κB pathway might be involved in the reduced renal inflammation in db/db mice after simultaneous blocking of VEGF-B and IL-17A

**Fig. 5:**
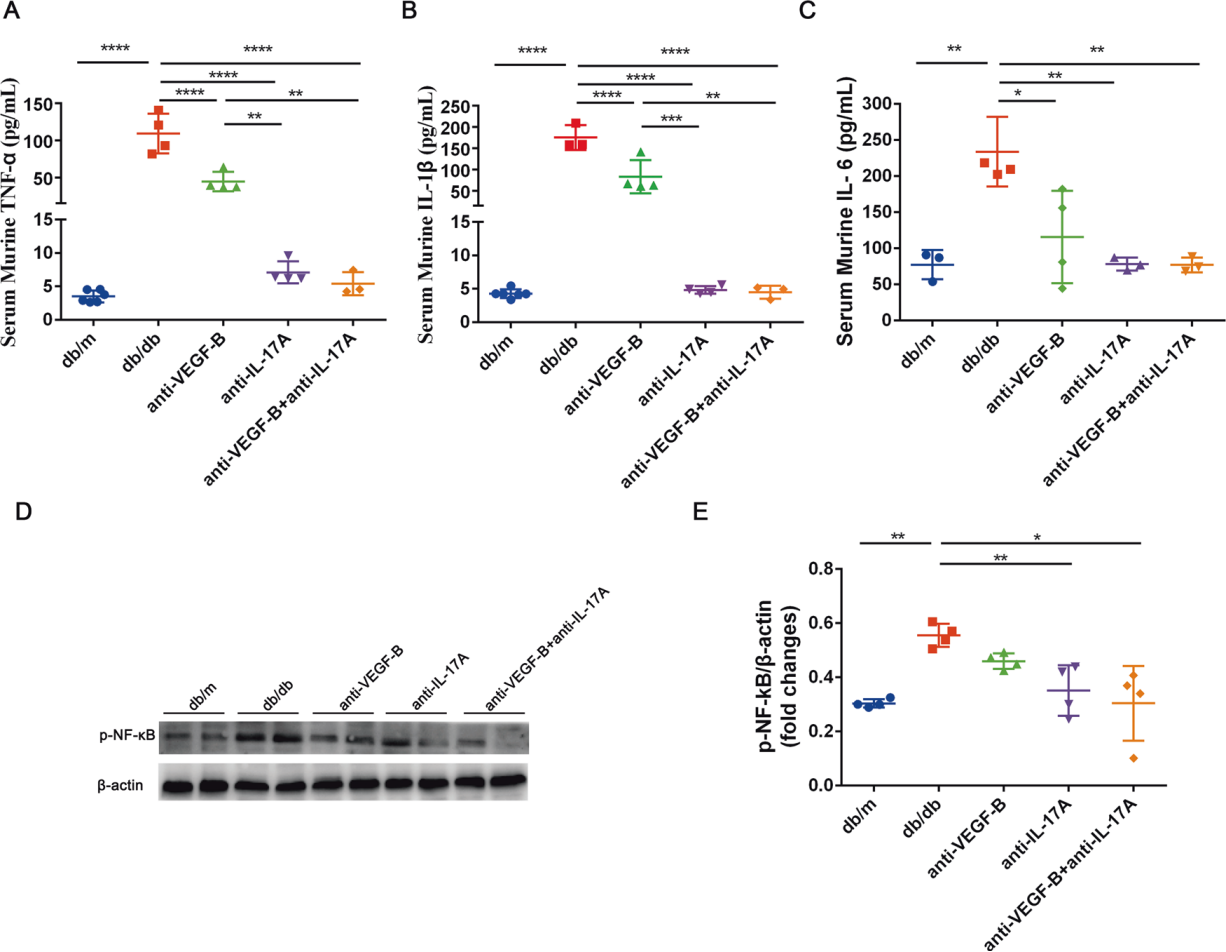
Pharmacological neutralization of VEGF-B and IL-17A reduced the renal inflammation in DKD mouse model. **A** The content of TNF-α in serum was measured by ELISA. **B** The content of IL-1β in serum was measured by ELISA. **C** The content of IL-6 in serum was measured by ELISA. **D** Western blot analysis of NF-κB p65 in all groups of mice. **E** Quantitative analysis of NF-κB p65 protein expression. The data were presented as means ± SD (for ELISA assay, *n* = 3–6 mice in each group; for the analysis of NF-κB p65, *n* = 2 mice in each group, data were representative of two independent experiments, ^****^P < 0.0001, ^**^P < 0.01, ^*^P < 0.05). The statistical significance was analyzed by one-way ANOVA with Tukey’s multiple-comparisons test.

It was reported that high levels of proinflammatory cytokine production were correlated with the progression of DKD [17]. Therefore, the anti-inflammatory effect of the combination of anti-VEGF-B and anti-IL-17A therapy in protecting renal function was investigated. The results indicated that serum cytokine productions of TNF-α (Fig. 5A), IL-1β (Fig. 5B), and IL-6 (Fig. 5C) were significantly elevated in db/db mice. Anti-VEGF-B or anti-IL-17A therapy lowered the expression of TNF-α, IL-1β, and IL-6 in db/db mice, the combined treatment of anti-VEGF-B and anti-IL-17A almost normalized the content of proinflammatory factors TNF-α, IL-1β and IL-6.

We also found that NF-κB p65 protein expression was downregulated after anti-VEGF-B and anti-IL-17A treatment (Fig. 5D, E). Furthermore, we analyzed the changes of gene expression levels of NF-κB components in db/m mice, db/db mice and combination-treated mice (Supplementary Fig. 8). In the KEGG NF-κB signaling pathway background, the majority of canonical NF-κB signaling pathway genes were upregulated in db/db mice and were reversed by the combination treatment (Supplementary Fig. 8A). Zooming in into individual genes (e.g. Nfkb1, Tnf, Traf6, il1r1), they tended to be upregulated in db/db mice, while no statistical difference was observed between combination treatment and db/m mice, which suggested that the combination treatment reversed this upregulation trend in canonical NF-κB signaling pathway genes in db/db mice (Supplementary Fig. 8B–E). This evidence suggested that the simultaneous blockade of VEGF-B and IL-17A in db/db mice could regulate the NF-κB pathway which might be involved in kidney injury in DKD.

Taken together, inhibition of the NF-κB pathway might be involved in the reduced renal inflammation after treatment of db/db mice with the neutralizing VEGF-B and IL-17A antibodies.

### The combined administration of anti-VEGF-B and anti-IL-17A reversed DKD-related transcriptomics changes

**Fig. 6:**
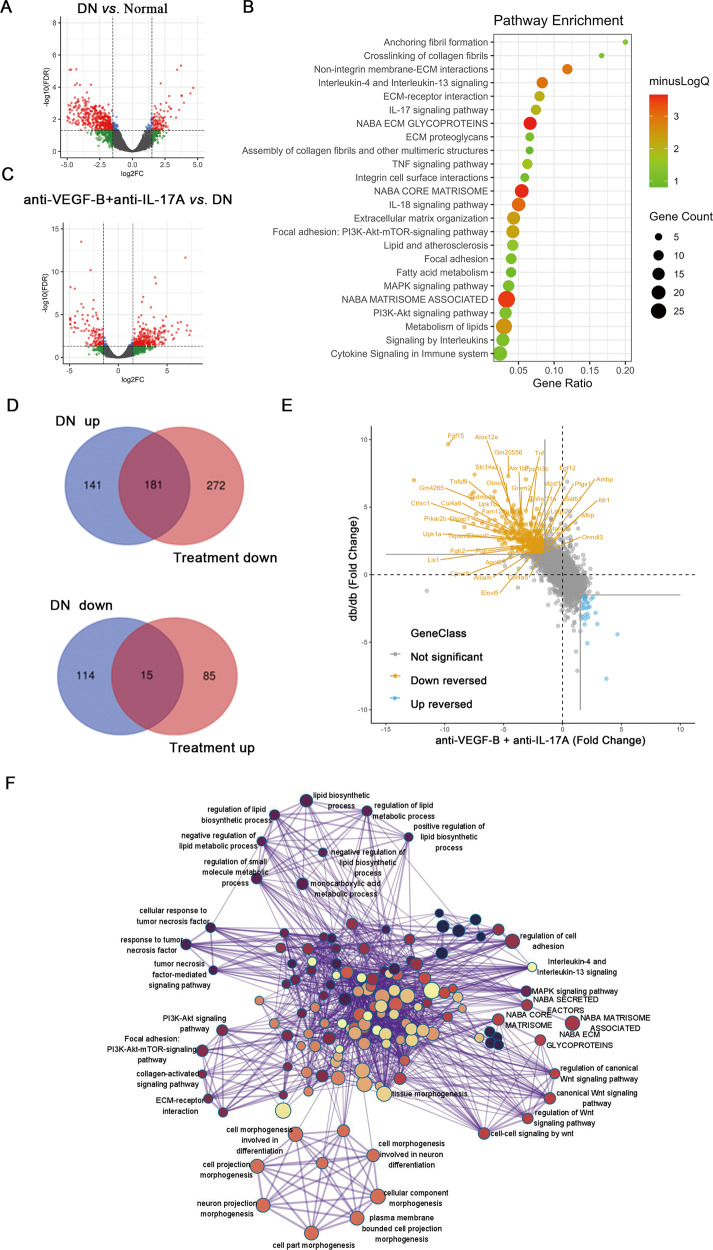
Transcriptomics analysis of effects of the combined treatment of anti-VEGF-B and anti-IL-17A on DKD mice. **A** DEGs analysis results were presented in volcano plot to show the genes of upregulation and downregulation in db/db (DKD) mice versus db/m (normal) mice. **B** Pathway enrichment analysis of 322 upregulated genes in DKD mice (compared with db/m normal control). Bubble size and color density indicated the number of enriched genes and -log10 (FDR-adjusted P-value) of enrichment analysis, respectively. **C** DEGs analysis results were presented in volcano plot to show the upregulated and downregulated genes in combination treated versus untreated db/db mice. **D** Venn diagrams showed the overlaps between “db/db-up” DEGs (upregulated DEGs in db/db mice compared with db/m mice) and “Treatment-down” DEGs (downregulated DEGs in treated db/db mice with anti-VEGF-B plus anti-IL-17A, compared with untreated db/db mice) and overlaps between “db/db-down” and “Treatment-up” DEGs. Overlapped DEGs mainly concentrated in “db/db-up” and “Treatment-down” DEGs comparison, which led to 181 upregulated DEGs in the DKD model that were reversed by the combination of anti-VEGF-B plus anti-IL-17A. **E** Gene expression fold changes (in log2 (fold-change) unit) in db/db versus db/m mice comparison (*y*-axis) and in treated db/db mice versus in untreated db/db mice comparison (*x*-axis) were visualized in a 2-dimensional scatter plot. Solid lines in gray color indicated the fold-change cutoffs to define DEGs. Scatter points in orange color located in the left-up region indicated these down-reversed 181 DEGs selected in (**D**). Representative genes which were closely related to lipid metabolism, inflammation, and renal fibrosis were labeled with gene name. **F** Network visualization of enriched GO terms and pathways of enrichment analysis of 181 down-reversed DEGs. Representative GO terms and pathways were labeled with specific names.

To uncover underlying mechanisms of the therapeutic effect of anti-VEGF-B and anti-IL-17A in DKD mice, the transcriptomic changes of mice renal tissues were measured using RNA-seq. As shown in Fig. 6A, RNA-seq analysis identified 451 DEGs between db/m and db/db mice. Among them, 322 genes were transcriptionally upregulated, 129 genes were downregulated. Pathway enrichment analysis indicated that 322 upregulated DEGs are mainly clustered into several signaling groups: metabolism, inflammation, fibrosis and other DKD-related pathways (Fig. 6B). In line with the findings from DKD patient dataset (Fig.1) and other literatures [28, 29], this result recapitulated that abnormal lipid metabolism and disorganized inflammation were heavily involved in the progression of DKD.

There were 553 DEGs (453 upregulated and 100 downregulated) between db/db mice and treated with anti-VEGF-B plus anti-IL-17A mice (Fig. 6C). Importantly, the combined treatment of anti-VEGF-B and anti-IL-17A down-reversed expression changes of 181 genes which were upregulated in DKD, as shown in the intersection area in Venn diagrams of Fig. 6D and the up-left region of Fig. 6E (full gene list was supplied in Supplementary Table 3). To probe whether this reversion of gene expression changes was mechanistically informative, we conducted GO and pathway enrichment analysis. Hundreds of GO terms and pathways were remarkably enriched by these 181 genes (full enrichment list could be found in Supplementary Table 4). To better visualize the enrichment results, enriched items were clustered according to their gene membership similarity and then converted to a network layout. As shown in Fig. 6F, these reversed genes were enriched in lipid metabolism, inflammation, and renal fibrosis-related biological processes and signaling pathways, where representative GO biological processes and pathways were highlighted with text labels. Zoom into details, these enriched items could be summarized into several categories: lipid biosynthesis and metabolism, inflammation regulation, fibrosis, cell morphogenesis, etc. Interestingly, among them, we found that fibroblast growth factor (FGF) family genes such as Fgfr2 and Fgf12 were upregulated in db/db mice and down-reversed obviously in the combined treatment, and the down-reversion might be mediated by the PI3K-Akt signaling pathway.

## Discussion

Currently, clinical treatments of DKD mainly rely on the control of traditional risk factors including hyperglycemia and elevated blood pressure [30]. However, these therapeutics could not effectively ameliorate the progression of DKD and reduce the risk of ESRD in some patients [31]. Although several new anti-diabetic drugs were found to possess notable renal protective effects [32], there were limited effective therapeutic approaches to control DKD progression before the kidney damage became irreversible. Therefore, keeping on trying to explore the pathogenesis of DKD and developing novel and effective therapies are urgently needed.

It had been reported that the VEGF-B signaling pathway was activated in DKD mice and patients [5, 22] and VEGF-B-mediated lipid deposition in kidney tissue might drive DKD development [5]. It implied that VEGF-B would be a potential therapeutic target for DKD treatment. VEGF-B controlled renal ectopic lipid deposition by regulating endothelial fatty acid transport [5, 10]. In addition, the inhibition of VEGF-B signaling by either gene knockout or anti-VEGF-B neutralizing antibody could prevent lipid deposition in DKD [5, 29]. Consistently, we also verified that anti-VEGF-B reduced the lipid droplets, especially the neutral lipids in the kidney in the DKD mouse model. Currently, a humanized anti-VEGF-B monoclonal antibody CSL346 is undergoing a phase 2 clinical trial to assess efficacy, safety, tolerability and pharmacokinetics for DKD treatment (ClinicalTrials.gov Identifier: NCT04419467). Although anti-VEGF-B antibodies could reduce lipid deposition and improve renal lipid metabolism, the renal injury resulting from long-term lipotoxicity is hard to be self-repaired. We and others had revealed that IL-22 was beneficial for accelerating kidney recovery and regeneration [33–35]. Our study revealed that cytokine IL-22 exhibited synergistic efficacy with anti-VEGF-B to ameliorate DKD progression [22]. Interestingly, transcriptomics analysis in this study indicated that the IL-17A signaling pathway is significantly enriched in db/db mice treated with the anti-VEGF-B-IL-22 fusion protein. We, therefore, hypothesized that the IL-17A signaling pathway is important for the effectiveness of VEGF-B-based DKD therapy.

Indeed, accumulating evidence showed that inflammation was a cardinal pathogenetic factor of DKD [11]. And importantly, IL-17A is such a cytokine that exhibits both critical pathogenic relevance in DKD [17, 18, 23] and druggable properties for development [36]. It participates in the pathogenesis of different autoimmune and inflammatory diseases [17, 37]. Three IL-17A targeted monoclonal antibodies have been approved for psoriasis, psoriatic arthritis (PA) and ankylosing spondylitis (AS) treatment [36]. Although anti-IL-17A antibody has not been evaluated for DKD treatment, recent research demonstrated that IL-17A was involved in diabetes-mediated renal damage [17, 18, 23]. The IL-17A-deficient mice or blockade of IL-17A with neutralizing antibody ameliorated renal dysfunction and protected against progression in DKD mice by inhibiting the renal inflammatory response [18, 19]. This evidence highlighted the critical roles of IL-17A signaling in renal damage and its potential clinical value as a therapeutic target for DKD treatment.

Therefore, in the current study, we attempted to develop a novel therapy that combines anti-VEGF-B and anti-IL-17A antibodies for DKD treatment. To this end, we firstly confirmed that both renal lipid metabolism abnormality and inflammation were closely involved in the progression of DKD by performing bioinformatics analysis on gene expression profiles from 112 DKD patients. Further analysis revealed that VEGF-B and IL-17A signaling pathways acted as critical players to cooperatively contribute to DKD onset. To test if simultaneous blockade of these two pathways could benefit to DKD treatment, we developed anti-VEGF-B and anti-IL-17A neutralizing antibodies and evaluated their efficacy on db/db mice. In theory, this combination therapy would not only reduce the renal ectopic lipid deposition by anti-VEGF-B blockage but also relieve the inflammatory response by blocking the IL-17A pathway. As expected, the results confirmed that simultaneous blockade of VEGF-B and IL-17A could ameliorate renal dysfunction by decreasing renal lipid accumulation and inflammatory response, they also alleviated the renal fibrosis in vivo. Blockade VEGF-B and IL-17A had no influence on the body weight, we observed the fat pads accumulated in the subcutaneous tissue of the abdomen in the dissected mice after combination treatment for 8 weeks. We speculated that the fat pads might be caused by the lipids which were shunted to the white adipose tissue. The IPGTT results displayed no significant differences in glucose tolerance in all treated groups, indicating that the beneficial effect of the combination therapy on renal function might not be ascribed to glycemic control. The result was in line with the finding that renal overexpression of VEGF-B was not the inducer of hyperglycemia [5].

To uncover the underlying mechanisms of the therapeutic effect of anti-VEGF-B plus anti-IL-17A on DKD mice, RNA-seq transcriptomic profiling was performed. The upregulated DEGs in db/db mice were mainly clustered into lipid metabolism (such as Fat4, Elovl5, Apold1, Grem2, Stard13), IL-17A-dependent inflammation (Cxcl1, Tnf, Tnfsf9, Tnfrsf11a, Nfκbia, Nfkb2, Cpne5, Tspan6), fibrosis (Col4a5, Fgf12, Fgf15) and other DKD-related pathways indicating that changes at transcriptomics level captured the pathomechanism information about DKD. However, the RNA-seq analysis failed to conclude statistically significant changes of expression levels of Vegfr-1, Fatp3, Fatp4 and Il17ra genes after anti-VEGF-B and anti-IL-17A treatment (Supplementary Fig. 9A–D). This might be due to the low sample size (*n* = 3) of our experiments and the relatively low signal-to-noise ratio of individual genes in high-throughput profiling data. Therefore, we observed the overall changes and the underlying biological meanings at gene list level, rather than individual genes, to probe the underlying mechanism of this combinatory treatment from the system level. The combinatory treatment remarkably reversed 181 genes which were significantly changed in DKD mice, and importantly, these genes were significantly overrepresented in pathways and biological processes that were closely related to lipid metabolism, inflammation, and renal fibrosis, based on the pathway and GO enrichment analysis. Interestingly, FGF family genes such as Fgfr2, and Fgf12 were down-reversed obviously in combined treatment, and the down-reversion might be mediated by the PI3K-Akt signaling pathway.

**Fig. 7:**
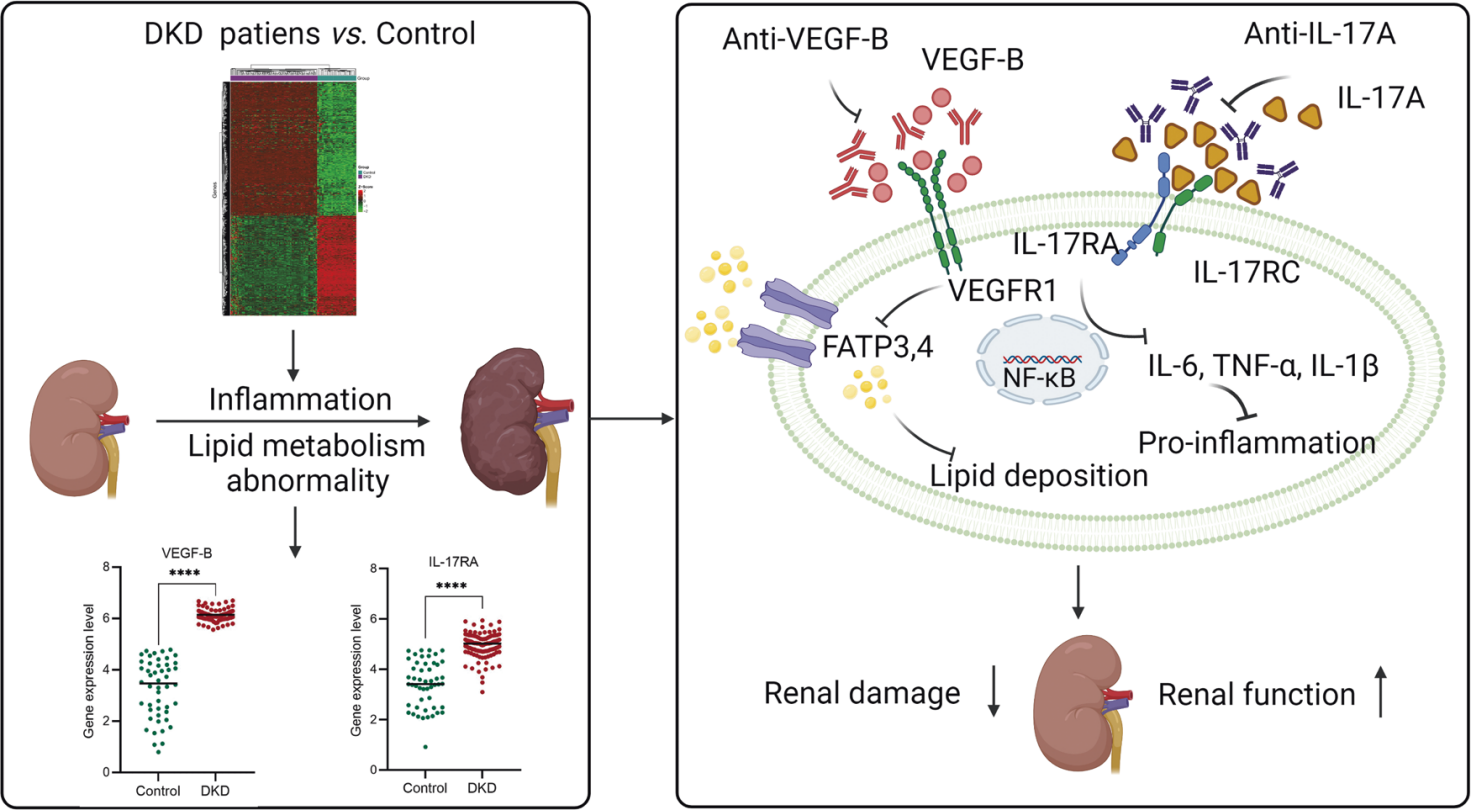
The graphical abstract of neutralizing of VEGF-B and IL-17A simultaneously to alleviate renal damage and ameliorate renal function. It was demonstrated that the renal lipid metabolism abnormality and inflammation significantly changed in DKD condition by mining public transcriptomic data of DKD patient samples. Further analysis exhibited the critical role of VEGF-B and IL-17A signal pathways in DKD progression, it indicated that VEGF-B and IL-17A might be promising targets for DKD treatment. Then the potential of a novel combination therapy, anti-VEGF-B plus anti-IL-17A antibody, was evaluated for DKD treatment. The results demonstrated that simultaneous blockade of VEGF-B and IL-17A signaling with neutralizing antibodies exhibited a synergistic effect on alleviating renal injury and ameliorating the renal function by reducing lipid deposition, normalizing the content of proinflammatory and alleviating the renal fibrosis in the DKD mouse model.

Taken together, in this study we firstly demonstrated that the renal lipid metabolism abnormality and inflammation significantly changed in DKD condition by mining public transcriptomic data of DKD patient samples (Fig. 7). Further analysis exhibited the critical role of VEGF-B and IL-17A signal pathways in DKD progression, it indicated that VEGF-B and IL-17A might be the promising targets for DKD treatment. Simultaneous blockade of VEGF-B and IL-17A signaling using neutralizing antibodies exhibited a synergistic effect on alleviating renal injury and ameliorating the renal function by decreasing lipid deposition, normalizing the proinflammatory content and preventing the renal fibrosis in the DKD mouse model, suggesting that simultaneous blockade of VEGF-B and IL-17A representing a novel effective therapy for DKD treatment.

## Materials and methods

### Publicly available DKD gene expression data collection

We searched GEO database using “diabetic kidney disease” as a keyword and only kept studies labeled as “*Homo sapiens*” organisms. Detailed metadata of samples from searched studies were downloaded from GEO website and then subjected to manual inspection to select qualified samples: (1) removed samples from patients with other disease conditions, kept samples from normal condition or DKD condition; (2) only kept samples derived from human kidney tissue; and (3) only kept samples that were measured gene expression profiles by microarray or RNA-seq assays and the expression data were available on GEO website. After removing duplicates, these steps led to a human DKD gene expression dataset “hDKD-expression dataset” including 50 normal kidney samples and 112 DKD kidney samples from 6 independent studies.

### Publicly available DKD gene expression data preprocessing and analysis

Rank-In algorithm [38], a state-of-the-art method for genomics data integration, was used to integrate gene expression profiles of our collected samples. Specifically, the “series-matrix” file of each study was downloaded from GEO and gene IDs in the original files were converted to gene symbols according to the annotation files of each profiling platform. We only kept genes that were shared between profiling platforms of these studies (9823 genes) and combined samples to a gene expression matrix (162 samples*9823 genes). The gene expression matrix as well as the sample group label (Control/DKD) and gene expression platform label were submitted to the Rank-In web server. Rank-In web server returned a normalized gene expression matrix which was used in downstream analysis. The DEGs analysis was performed using limma (v3.48.3) R package [39] with a threshold of |log2FC | ≥ 1.5 and FDR-adjusted *P* value ≤ 0.01. Gene Ontology (GO) enrichment analysis of the DEGs gene list was performed by the Metascape web server [40].

### Mouse renal tissue RNA-seq profiling and bioinformatics analysis

The renal cortical samples from the db/m group, db/db group, anti-VEGF-B and anti-IL-17A-treated group (*n* = 3) were collected and the total RNA was extracted. The integrity and purity of RNA were detected by Agilent 2100 Bioanalyzer system (Agilent, Santa Clara, CA). All RNA samples with RIN ≥ 8 were considered high‐quality. The samples were sequenced with the Illumina NovaSeq 6000. Mouse genome reference was built using GRCm38 (mm10) version by STAR (v2.7.1a) [41]. Raw sequencing data in fastq format was analyzed by the quality control pipeline in fastqc and then mapped to mouse genome reference by using STAR with default parameters. Subsequently, read counts of genes were quantified by the featureCounts module of the Rsubread (v2.0.0) R package for downstream analysis [42]. The significantly modified genes were selected based on differential expressed gene (DEG) analysis, which was performed using edgeR (v3.34.1) R package with the threshold of |log2FC | ≥ 1.5 and FDR-adjusted *P* value ≤ 0.05 to define significantly upregulated and downregulated genes between compared groups. DEGs gene list was submitted to the Metascape webserver for GO enrichment analysis [40]. Gene expression levels in Transcripts Per Million (TPM) mapped reads were quantified by using Kallisto (v0.46.1) R package [43]. In order to visualize the changes in gene expression levels of the KEGG signaling pathway in db/m mice, db/db mice, and anti-VEGF-B plus anti-IL-17A treated mice, the standard pathway was used as the template. The gene expression values in TPM format were firstly transformed into *Z*-score, the averaged *Z*-scores of all genes in each group were subsequently calculated and submitted to the Pathview web server [44]. In the resulting pathway view image, red color represented relative upregulated expression and green represented relative downregulated expression.

### Cell culture

HUVEC, NIH-3H3 cells were acquired from the Cell Bank of the Chinese Academy of Sciences, Shanghai Branch (Shanghai, China). The cells were cultured in DMEM medium with 10% of fetal bovine serum (FBS), 2 mM l-glutamine and 1% penicillin–streptomycin and maintained in a humidified incubator (37 °C, 5% CO_2_). The DMEM, FBS and other regents were purchased from Gibco, Thermo Fisher Scientific Inc. The cell lines were authenticated by the Cell Bank of the Chinese Academy of Sciences and confirmed mycoplasma-free.

### Expression, purification and evaluation of anti-VEGF-B and anti-IL-17A

The plasmids of anti-VEGF-B (2H10) [45] and anti-IL-17A (Vunakizumab) were developed and then transfected transiently into Chinese hamster ovary (CHO) cells for the expression the antibodies. The supernatants were collected and then the proteins were purified. The purity of neutralizing antibodies was characterized by high-performance size-exclusion chromatography (HPSEC). The bioactivity of anti-VEGF-B was performed to test the expression of FATP4 which was inhibited by blocking the interaction of VEGF-B and its receptor VEGFR-1 in HUVEC cell lines by using western blot, the bioactivity of anti-IL-17A was performed to measure the production of IL-6 inhibited by blocking the interaction of IL-17A and its receptor IL-17RA in NIH-3H3 cell lines with ELISA assay. The specificity was determined by analyzing the interaction of anti-VEGF-B and VEGF-B, anti-IL-17A and IL-17A based on the surface plasmon resonance (SPR) technique. Histological analysis of heart, liver, spleen, lung and kidney tissues was conducted to evaluate the toxicity of anti-VEGF-B and anti-IL-17A in vivo.

### Animal models and treatment

Six-week-old male db/db mice (C57BLKS/J-Lepr^em2Cd479^/Gpt) and wild-type db/m mice were from GemPharmatech Co., Ltd (Nanjing, China). They were kept in specific pathogen-free grade conditions, the light/dark cycle was 12/12 h, the temperature was controlled between 20 and 24 °C. The animal study in this research was approved by the Animal Ethical Committee of the School of Pharmacy. The research methods applied in this study met the criterion of the approved regulations and guidelines.

The db/m mice were fed with chow food and used as the control group (*n* = 7), the db/db mice have kept a high-fat diet (HFD) throughout the animal experiment and were randomly divided into four groups: db/db group (DKD model, *n* = 9), anti-VEGF-B or/and anti-IL-17A treated groups (*n* = 7, each group). After the DKD model was established, the mice were then treated with anti-VEGF-B or/and anti-IL-17A by intraperitoneal (i.p.) injection twice a week for 8 consecutive weeks. The dosage was 0.4 mg anti-VEGF-B or/and 0.2 mg anti-IL-17A per animal.

### Blood, urine and tissue sample collection

Mice blood samples were collected from the orbital venous plexus. Urine was collected by housing mice in metabolic cages which were sterilized before use to avoid contaminant interferences. Mice blood and urine samples were collected once every week from 8 weeks old mice to monitor the blood glucose, urinary microalbumin and urinary creatinine until the DKD model was established. Then the mice blood and urine samples were collected once every two weeks after the administration of anti-VEGF-B or/and anti-IL-17A. The body weight was measured once a week. After 8 weeks of treatment, the mice were sacrificed and kidney tissues were harvested and frozen for further analysis.

### Metabolic measurements

The creatinine was determined by the Creatinine (Cr) Assay Kit. Urine microalbumin was quantified by the Mouse Microalbumin ELISA Kit. The microalbumin to creatinine ratio (ACR) was determined by the ratio of the urinary microalbumin to the urinary creatinine. Creatinine clearance rate (Ccr) was calculated by the equation: Ccr = (urine creatinine × urine volume/24 h)/serum creatinine. The renal index (RI) was determined by the ratio of kidney weight to body weight, it is an objective, reproducible tool to predict renal function [46]. Postprandial blood glucose levels were measured once every 2 weeks employing the Glucose Assay Kit. Intraperitoneal glucose tolerance test (IPGTT) was performed after the mice fasted for 12 h. The serum triglyceride and free fatty acids (FFAs) were also measured by the Triglyceride Assay Kit and Nonesterified Free Fatty Acids Assay Kit (Nanjing Jiancheng Bioengineering, Nanjing, China).

### Histopathological analysis

In order to evaluate the histopathological features of the kidney, the renal tissues were fixed with 4% formaldehyde and sectioned at 5 µm thickness, and then embedded in paraffin and stained, or the renal tissues were made into frozen sections. 3–4 mice were randomly selected from each group and per section per mice was analyzed. Hematoxylin and eosin (H&E) staining was used to evaluate the renal histopathological damage. Periodic acid-Schiff (PAS) staining was conducted for the glomerulus mesangial expansion (GME) evaluation by the glomerular mesangial index. Masson staining was used to evaluate the collagen deposition in renal tissues. Oil-red O staining was performed to assess the neutral lipids in the frozen tissue section. They were visualized by SlideView VS200 (Olympus, Japan). The percentages of positive PAS, Masson, Oil red O stained area in the whole image were quantified with the Image J software (NIH, Bethesda, MA, USA).

### Immunohistochemical analysis

The expressions of Fibronectin and α-SMA were determined by the immunohistochemical staining with Fibronectin Rabbit mAb (Abcam, Cambridge, UK, ab268020) and α-SMA Rabbit mAb (Abcam, Cambridge, UK, ab108424). DAPI (Abcam, Cambridge, UK, ab285390) was used to counterstain the nuclear DNA. The images were taken by SlideView VS200 (Olympus, Japan). The percentages of positive Fibronectin and α-SMA stained area in the whole image were quantified with the Image J software (NIH, Bethesda, MA, USA).

### Immunofluorescence

ADRP is a reliable lipid droplet marker and plays an important role in intracellular lipid metabolism [47–49]. The expression of adipophilin (ADRP) in the kidney sections was determined with immunofluorescence staining, it was stained with an anti-ADRP primary antibody (CST, CA, USA, #45535) and the secondary antibody was the Goat anti-Rabbit IgG H&L (Alexa Fluor® 488) (Abcam, Cambridge, UK, ab150077), DAPI was used to counterstain the nuclear DNA. The images were taken by confocal microscopy (LSM 700, Carl Zeiss Inc., Germany). The percentages of positive relative fluorescence intensity in the whole image were calculated.

### Transmission electron microscopy analyses

Three mice were randomly selected from each group, and the renal tissue samples were prepared with 4% stationary liquid and were observed under transmission electron microscopy at 80 kV. And the thickness of the glomerular basement membrane was measured. The number of podocyte slits per micrometer (slit density) was calculated.

### Western blot analysis

The cell suspension was prepared from the kidney tissue homogenate and then was lysed with RIPA buffer. The total protein was extracted and determined the concentration. The expression of NF-κB p65 was tested by western blot with NF-κB p65 Rabbit mAb antibody (CST, CA, USA, #3033S). The expression of FATP4 protein was tested with FATP4 Rabbit mAb (Abcam, Cambridge, UK, ab199719). The internal reference protein antibodies were GAPDH Rabbit mAb (CST, CA, USA, #5174S) and β-actin Rabbit mAb (CST, CA, USA, #4970S). The protein bands were detected by the Fluor Chem M system (ProteinSimple, CA, USA).

### Cytokine assay

The ELISA assay was used to quantify the contents of TNF-α, IL-1β and IL-6 in murine serum. The assay kits were purchased from Dakewe Biotech Co., Ltd. (Shenzhen, China) and Fine Biotech Co., Ltd. (Wuhan, China).

### Statistical analysis

The data results were processed with Graph Pad Prism 6 (San Diego, CA, USA). Statistically significant differences were analyzed by Student’s *t*-test and one-way or two-way ANOVA with Tukey’s multiple-comparisons test. The statistical significances were presented by the ^****^P < 0.0001, ^***^P < 0.001, ^**^P < 0.01, ^*^P < 0.05.

## Supplementary information

Supplementary files

Original Data File

Checklist

## Data Availability

All data are available in the main text and the supplementary materials.
