# Supplementary material for: Simultaneous blockade of VEGF-B and IL-17A ameliorated diabetic kidney disease by reducing ectopic lipid deposition and alleviating inflammation response
Source: Cell Death Discov. 2023 Jan 16;9:8. doi: 10.1038/s41420-023-01304-5 (PMC9842640; doi:10.1038/s41420-023-01304-5)
Supplement: Supplementary file 1 — Supplementary files [file 41420_2023_1304_MOESM1_ESM.docx]

**Supplemental Material**

**Supplementary Figure Legends**

**
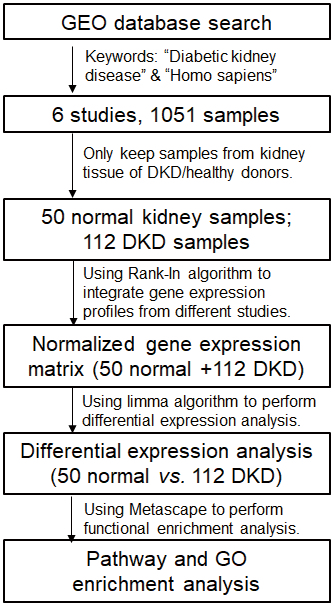
**

**Supplementary Fig. 1** **Workflow of collecting and analyzing publicly available gene expression samples from DKD patients.** We searched GEO database using “diabetic kidney disease” as keyword and only kept studies labelled as “Homo sapiens”. Detailed meta data of samples from searched studies were downloaded from GEO website and then subjected to manually inspection to select qualified samples. First, we removed samples from patients with other disease conditions, kept samples from normal condition or DKD condition; Then we only kept samples derived from human kidney tissue and only kept samples that were measured gene expression profiles by microarray or RNA-seq assays and the expression data were available on GEO website. Finally, these steps led to a human DKD gene expression dataset “hDKD-expression dataset” including 50 normal kidney samples and 112 DKD kidney samples from 6 independent studies.


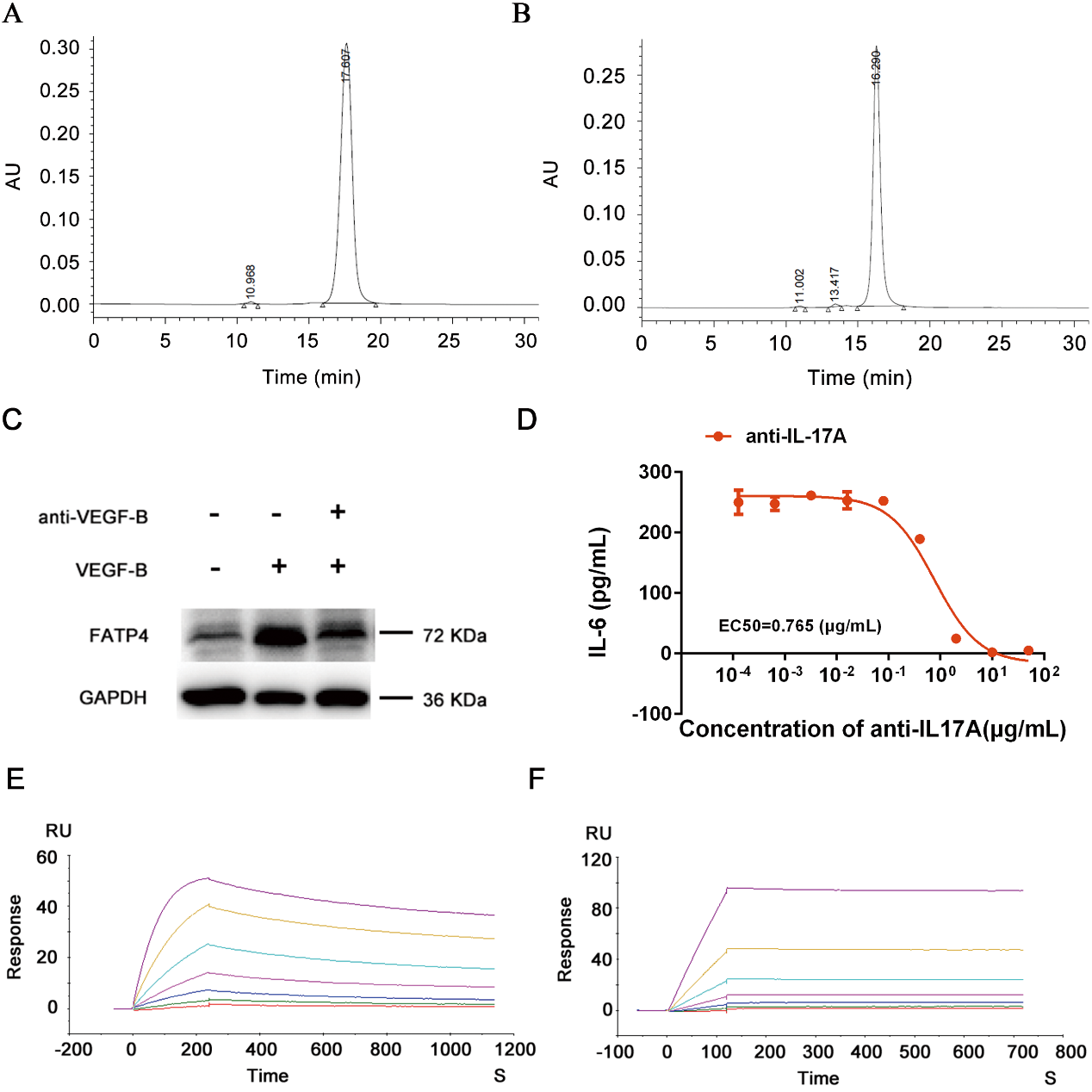


**Supplementary Fig. 2 The purity, activity and specificity of anti-VEGF-B and anti-IL-17A.**

**A-B** The purity of anti-VEGF-B and anti-IL-17A was characterized by high-performance size-exclusion chromatography. **C** The western blot analysis of the FATP4 expression which was inhibited by blocking the interaction of VEGF-B and its receptor VEGFR1 in HUVEC cells. **D** The IL-6 production inhibited by blocking the IL-17A signaling with anti-IL-17A in NIH-3T3 cells. **E** The surface plasmon resonance analysis was used to analyze the interaction between anti-VEGF-B and VEGF-B. **F** The surface plasmon resonance analysis was used to analyze the interaction between anti-IL-17A and IL-17A.


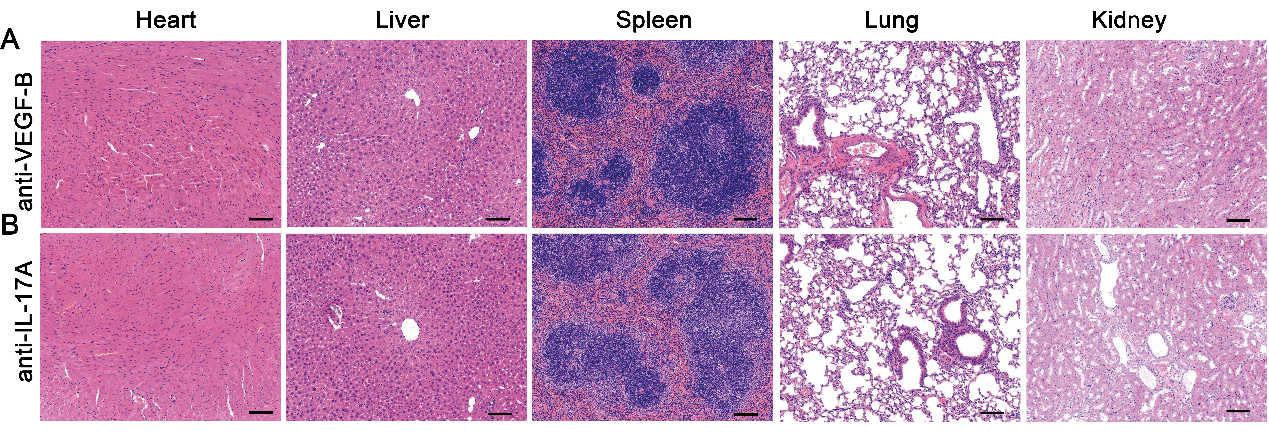


**Supplementary Fig. 3** **The toxicity of anti-VEGF-B and anti-IL-17A *in vivo* was evaluated.**

**A** Histological observation of mice heart, liver, spleen, lung, kidney after 7 days treatment of anti-VEGF-B. **B** Histological observation of mice heart, liver, spleen, lung, kidney after 7 days treatment of anti-IL-17A. n=3, Scale = 100 μm.


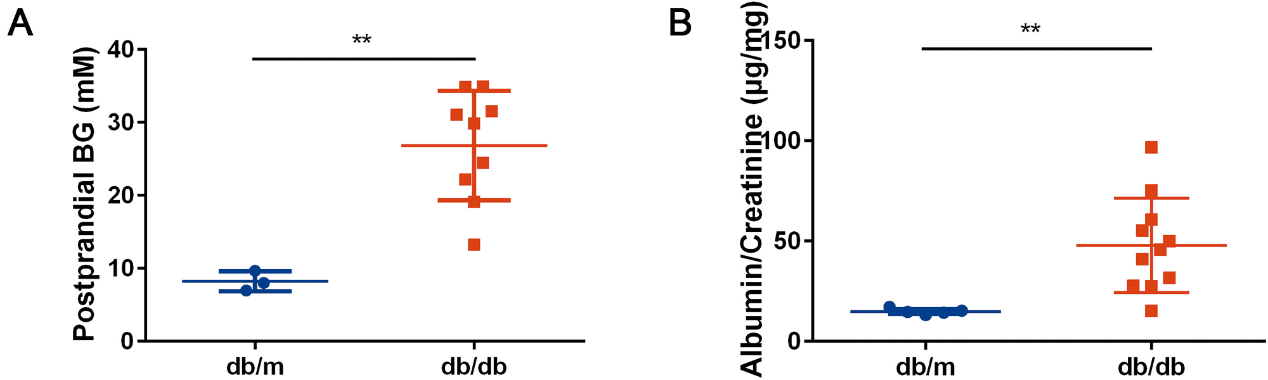


**Supplementary Fig. 4 The DKD mouse model was well established. A** The postprandial blood glucose in db/m and db/db mice at the age of ten weeks. **B** The ACR levels in db/m and db/db mice at the age of ten weeks. The data were presented as means ± SD, n=3-11 mice in each group, ^∗∗^*P* < 0.01 was determined by Student’s *t*-test between db/m group and db/db group.


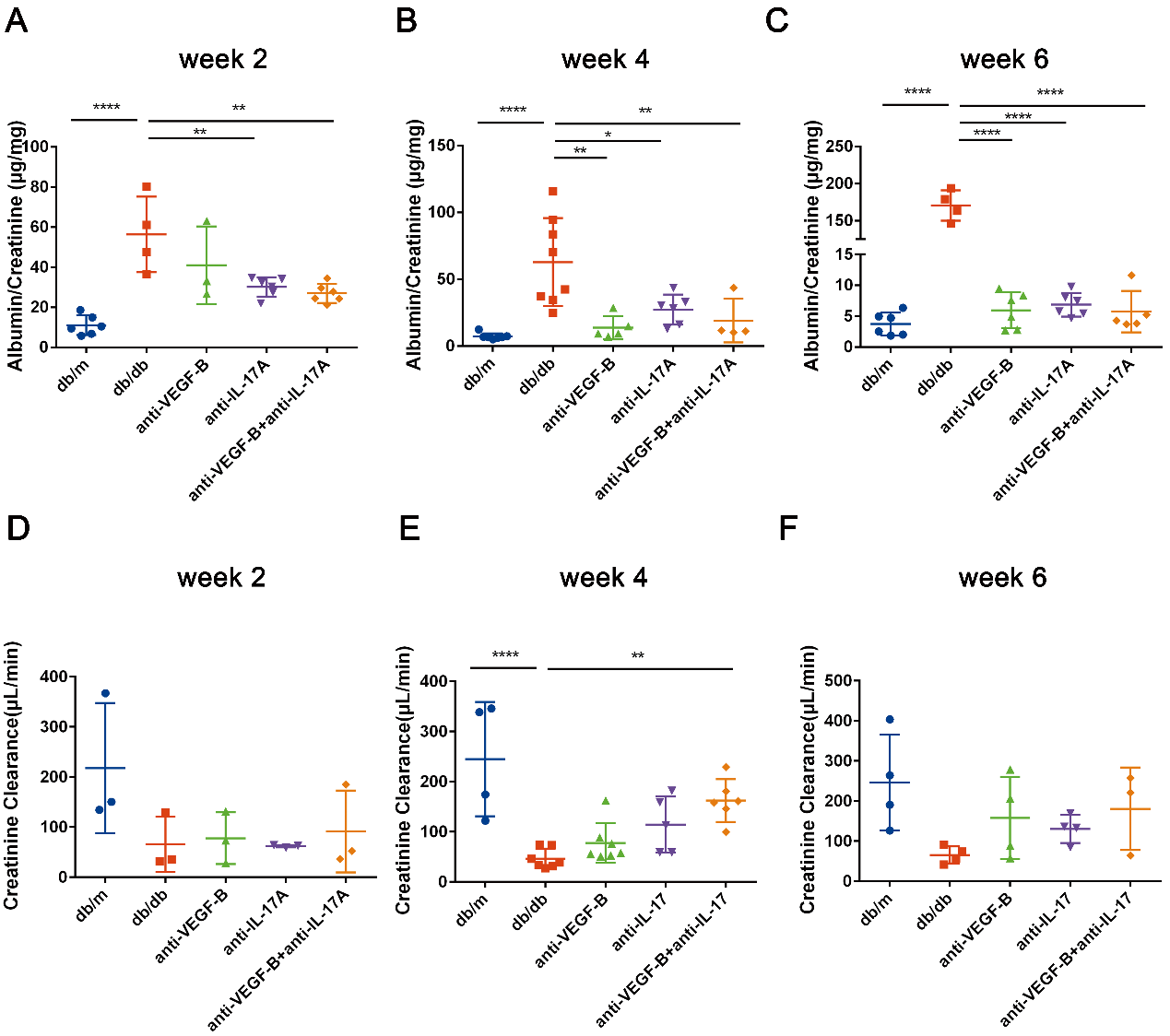


**Supplementary Fig. 5 The therapy effect of anti-VEGF-B or/and anti-IL-17A in DKD model mice for week 2, week4 and week 6. A**, **B** and **C** showed the ACR levels of anti-VEGF-B or/and anti-IL-17A treated mice for week 2, week 4 and week 6 with the HFD feeding. **D**, **E** and **F** showed the CCR levels of anti-VEGF-B or/and anti-IL-17A treated mice for week 2, week4 and week 6 with the HFD feeding. The data were presented as means ± SD, n=3-8 mice in each group, ^****^*P* < 0.0001, ^**^*P* < 0.01, ^*^*P* < 0.05. The statistical significance was analyzed by one-way ANOVA with Tukey’s multiple-comparisons test.


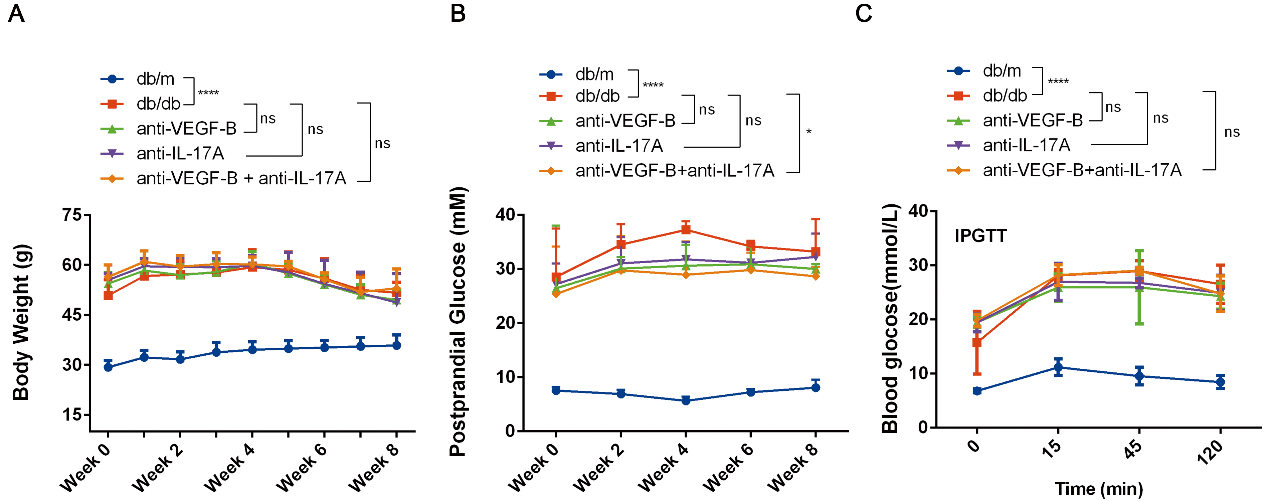


**Supplementary Fig. 6 Body weight, postprandial blood glucose, intraperitoneal glucose tolerance test of mice treated with anti-VEGF-B or/and anti-IL-17A. A** Measurement of body weight. **B** Measurement of postprandial blood glucose. **C** Measurement of intraperitoneal glucose tolerance test. The data were presented as means ± SD, n=3-6 mice in each group, ^****^*P* < 0.0001, ^***^*P* < 0.001, ^*^*P* < 0.05. The statistical significance was analyzed by two-way ANOVA with Tukey’s multiple-comparisons test.


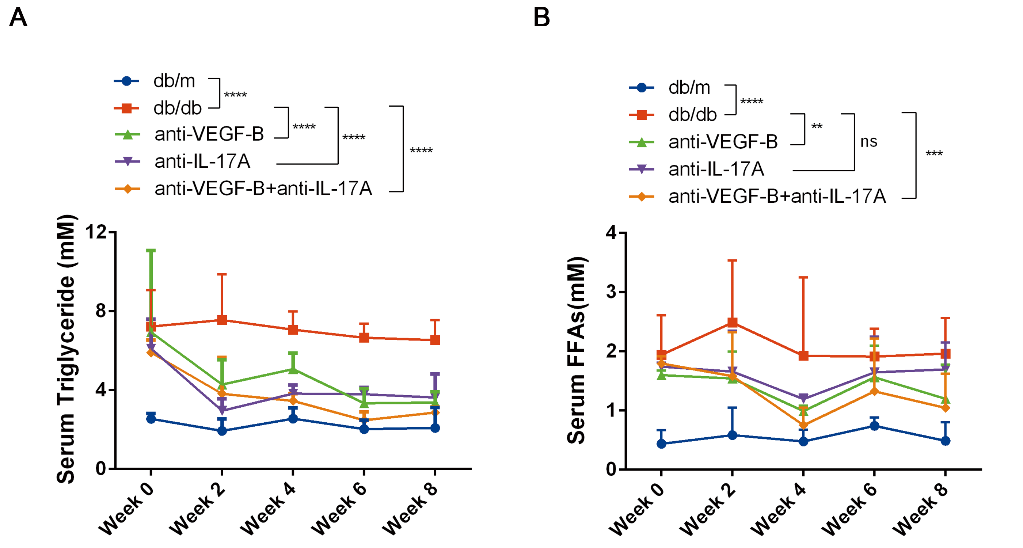


**Supplementary Fig. 7 Triglyceride and free fatty acids of mice treated with anti-VEGF-B or/and anti-IL-17A.** **A** Measurement of triglyceride. **B** Measurement of free fatty acids. The data were presented as means ± SD, n=3-7 mice in each group, ^****^ *P* < 0.0001, ^***^*P* < 0.001, ^**^*P* < 0.01. The statistical significance was analyzed by two-way ANOVA with Tukey’s multiple-comparisons test.


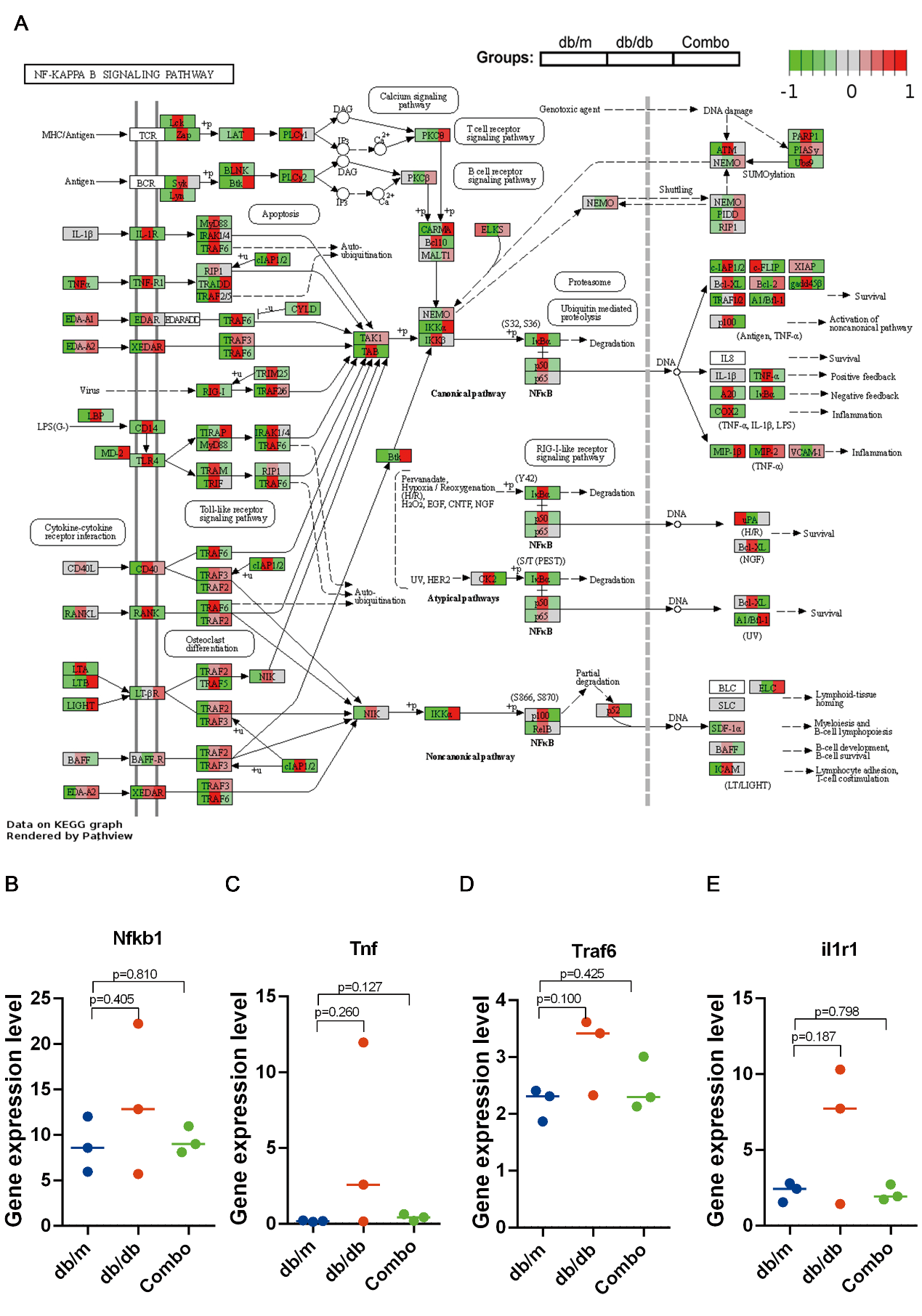


**Supplementary Fig. 8 Gene expression changes in components of NF-κB signaling pathway in db/m mice, db/db mice, and anti-VEGF-B plus anti-IL-17A treated mice. A** gene expression values (TPM) in all mice were transformed into Z-scores. The averaged Z-scores of individual genes in each group were visualized using Pathview webserver. Red color represented relative upregulated expression and green represented relative downregulated expression. **B-E** Expression levels (TPM) of representative genes were shown. **B** Nfkb1. **C** Tnf. **D** Traf6. **E** il1r1. Combo treatment is anti-VEGF-B and anti-IL-17A group, gene expression levels were quantified in TPM unit. *P* > 0.05 was determined by Student’s *t*-test, n = 3.


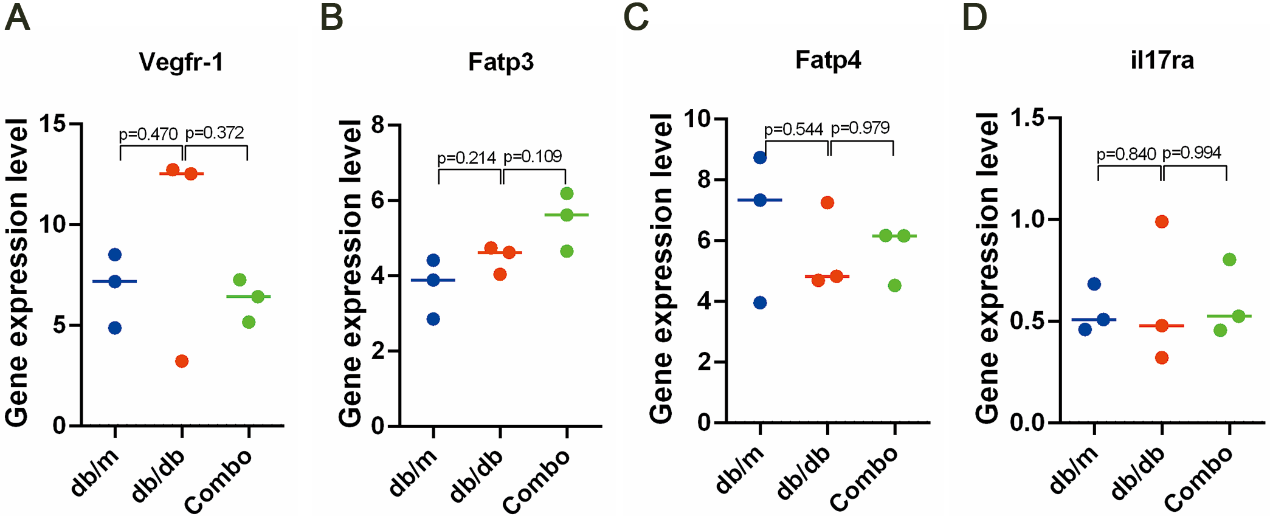


**Supplementary Fig. 9 Expression levels of Vegfr-1, Fatp3, Fatp4 and Il17ra genes in db/m group, db/db group, anti-VEGF-B plus anti-IL-17A treated group.**

**A** Expression level of Vegfr-1 gene in each group. **B** Expression level of Fatp3 gene in each group. **C** Expression level of Fatp4 gene in each group. **D** Expression level of Il17ra gene in each group. Combo treatment is anti-VEGF-B and anti-IL-17A group, gene expression levels were quantified in TPM unit. *P* > 0.05 was determined by Student’s *t*-test, n = 3.

**Supplementary Tables**

**Table 1. Publicly available gene expression samples from DKD patients and healthy controls.**

| **Study ID** | **Control samples** | **DKD samples** | **Gene expression profiling platform** |
| --- | --- | --- | --- |
| GSE30122 | 50 | 19 | GPL571 |
| GSE99339 | 0 | 14 | GPL19109; GPL19184 |
| GSE99325 | 0 | 18 | GPL19109; GPL19184 |
| GSE47185 | 0 | 32 | GPL11670; GPL14663 |
| GSE104948 | 0 | 12 | GPL22945; GPL24120 |
| GSE104954 | 0 | 17 | GPL22945; GPL24120 |
| Total | 50 | 112 | - |

**Table 2. Metascape pathway enrichment analysis of upregulated DEGs in DKD patients.**

| **Enrichment Data Source** | **Pathway Description** | **LogP** | **Log(q-value)** | **Enriched Gene Ratio** | **Enriched Genes** |
| --- | --- | --- | --- | --- | --- |
| WikiPathways | VEGFA-VEGFR2 signaling pathway | -17.4349 | -14.167 | 50/439 | ACACB,JAG1,AKT1,ARF6,CALR,CDH5,CREB1,ATF2,CYBB,S1PR1,ENG,EPRS1,ETS1,MTOR,GAB1,GATA2,ITGB3,LMAN1,MEF2C,PAK2,PDPK1,PRKCA,PRKCE,PRKCI,MAPK1,PTPN11,PTPRJ,RAB5A,CCL2,MAP2K4,SHC1,SLC7A1,SRF,PRDX2,TFCP2,TPM3,VCL,YWHAE,MAPKAPK5,SYNJ1,SH2D2A,TAOK2,MICAL2,CAMKK2,TRAF3IP2,STIP1,PRKD2,PBXIP1,ITCH,AMOT |
| WikiPathways | EGF/EGFR signaling pathway | -16.3573 | -13.289 | 30/163 | AKT1,ARF6,CAMK2A,CBLB,CREB1,CRKL,ERBB2,MTOR,GAB1,INPP5D,JUND,MEF2A,MEF2C,MAP3K1,PDPK1,PLD1,PRKCA,PRKCI,MAPK1,PTPN11,RAB5A,SHC1,SOS2,STAT5B,SYNJ1,SH2D2A,STAM2,MAP3K2,ASAP1,ITCH |
| Reactome Gene Sets | Signaling by Receptor Tyrosine Kinases | -16.3023 | -13.257 | 53/521 | AKT1,ARF6,ATP6V1A,ATP6V0A1,BAX,CDH5,COL1A1,COL3A1,COL6A3,CREB1,ATF2,CRKL,CYBB,ERBB2,FGFR1,MTOR,GAB1,ID1,INSR,ITGA2,ITGB3,JUND,LCK,MEF2A,MEF2C,NAB1,NCBP1,NCF4,NTRK3,PAK2,PAK3,PDPK1,POLR2E,PRKCA,PRKCE,MAPK1,MAPK11,PSEN2,PTN,PTPN11,PTPRJ,SHC1,SRF,STAT5B,TGFA,THBS1,THBS2,SH2D2A,ELMO1,STAM2,ATP6V0A2,ELMO2,ITCH,VCL,CAMKK2 |
| Reactome Gene Sets | Signaling by Rho GTPases, Miro GTPases and RHOBTB3 | -14.266 | -11.476 | 60/723 | ABL2,JAG1,AR,ARHGDIA,DST,CDH1,CHN2,CYBB,DBT,DOCK2,DVL3,SFN,LCK,LMAN1,NCF4,NUP98,PAK2,PAK3,PDPK1,PLD1,PPP2R5A,PRKCA,PKN2,MAPK1,MAPK11,RAC2,RANGAP1,ITSN1,SOS2,SPTBN1,SRF,TPM3,WAS,WIPF1,YWHAE,YWHAH,PICALM,BUB3,VAPB,IL32,ARHGEF10,GIT2,FLOT1,STAM2,SEMA4F,CCT2,GNA13,STIP1,FERMT2,AKAP13,RRAS2,TRAK1,NSL1,NCKIPSD,LRRC1,EMC3,ZNF512B,DOCK6,ELMO2,DOCK5 |
| Reactome Gene Sets | Signaling by Rho GTPases | -14.1416 | -11.363 | 59/707 | ABL2,JAG1,AR,ARHGDIA,DST,CDH1,CHN2,CYBB,DBT,DOCK2,DVL3,SFN,LCK,LMAN1,NCF4,NUP98,PAK2,PAK3,PDPK1,PLD1,PPP2R5A,PRKCA,PKN2,MAPK1,MAPK11,RAC2,RANGAP1,ITSN1,SOS2,SPTBN1,SRF,TPM3,WAS,WIPF1,YWHAE,YWHAH,PICALM,BUB3,VAPB,IL32,ARHGEF10,GIT2,FLOT1,STAM2,SEMA4F,CCT2,GNA13,STIP1,FERMT2,AKAP13,RRAS2,NSL1,NCKIPSD,LRRC1,EMC3,ZNF512B,DOCK6,ELMO2,DOCK5 |
| WikiPathways | Insulin signaling | -12.8604 | -10.238 | 26/161 | AKT1,ARF6,CBLB,MTOR,GAB1,INSR,MAP3K1,ENPP1,PDPK1,PRKCA,PRKCH,PRKCI,MAPK1,MAPK11,PTPN11,RAC2,RHEB,MAP2K4,SHC1,SLC2A1,SOS2,SRF,VAMP2,TSC2,FLOT1,MAP3K2 |
| Canonical Pathways | PID ERBB1 DOWNSTREAM PATHWAY | -12.3862 | -9.780 | 21/105 | AKT1,CHN2,CREB1,ATF2,MTOR,GAB1,SFN,SMAD1,MEF2C,MAP3K1,PDPK1,PLD1,PRKCA,MAPK1,RAB5A,MAP2K4,SRF,YWHAE,YWHAH,SH2D2A,MAP3K2 |
| Reactome Gene Sets | RHO GTPase cycle | -11.8081 | -9.281 | 42/449 | ABL2,JAG1,ARHGDIA,DST,CHN2,CYBB,DBT,DOCK2,LCK,LMAN1,NCF4,PAK2,PAK3,PLD1,PKN2,RAC2,ITSN1,SOS2,SPTBN1,TPM3,WAS,WIPF1,PICALM,VAPB,IL32,ARHGEF10,GIT2,FLOT1,STAM2,SEMA4F,CCT2,GNA13,STIP1,FERMT2,AKAP13,RRAS2,LRRC1,EMC3,ZNF512B,DOCK6,ELMO2,DOCK5 |
| Canonical Pathways | PID MET PATHWAY | -10.6891 | -8.306 | 17/79 | AKT1,ARF6,CDH1,CRKL,ETS1,MTOR,GAB1,MAP3K1,PAK2,PDPK1,PRKCI,MAPK1,PTPN11,PTPRJ,RAB5A,MAP2K4,SHC1 |
| Canonical Pathways | PID PDGFRB PATHWAY | -9.71652 | -7.449 | 20/129 | ARHGDIA,S1PR1,GAB1,SFN,ITGB3,JUND,LCK,PPP2R2B,PRKCA,PRKCE,MAPK1,PTPN11,PTPRJ,RAB5A,MAP2K4,SHC1,SRF,STAT5B,YWHAE,YWHAH |
| WikiPathways | Ras signaling | -9.10167 | -6.928 | 23/185 | ABL2,AKT1,ARF6,ETS1,FGFR1,GAB1,INSR,NF1,PAK2,PAK3,PLD1,PRKCA,MAPK1,PTPN11,RAB5A,RAB5B,RAC2,SHC1,SOS2,BRAP,RRAS2,MRAS,PLA1A |
| KEGG Pathway | Phospholipase D signaling pathway | -8.63571 | -6.525 | 20/148 | AKT1,ARF6,AVP,FCER1G,MTOR,GAB1,INSR,PLD1,PRKCA,MAPK1,PTPN11,RHEB,SHC1,SOS2,TSC2,DGKZ,GNA13,RRAS2,MRAS,PLCB1 |
| Canonical Pathways | PID VEGFR1 2 PATHWAY | -8.55895 | -6.465 | 14/69 | AKT1,CDH5,GAB1,ITGB3,PAK2,PDPK1,PRKCA,MAPK1,MAPK11,PTPN11,PTPRJ,VCL,SH2D2A,CAMKK2 |
| KEGG Pathway | Ras signaling pathway | -8.52211 | -6.433 | 25/232 | ABL2,AKT1,ARF6,EFNA5,ETS1,FGFR1,GAB1,INSR,NF1,PAK2,PAK3,PLD1,PRKCA,MAPK1,PTPN11,RAB5A,RAB5B,RAC2,SHC1,SOS2,TGFA,BRAP,RRAS2,MRAS,PLA1A |
| WikiPathways | Brain-derived neurotrophic factor (BDNF) signaling pathway | -8.0622 | -6.031 | 19/144 | ACACB,AKT1,CAMK2A,CREB1,MTOR,MARCKS,MEF2A,MEF2C,MAP3K1,NTRK3,PDPK1,MAPK1,PTPN11,SORT1,SHC1,STAT5B,TSC2,MAP3K2,SIRPA |
| KEGG Pathway | Chagas disease | -7.97866 | -5.958 | 16/102 | AKT1,C1QA,C1QB,CALR,CASP8,CD3E,NOS2,PPP2R2B,MAPK1,MAPK11,CCL2,MAP2K4,TGFBR2,CFLAR,GNA14,PLCB1 |
| WikiPathways | Angiopoietin-like protein 8 regulatory pathway | -7.90048 | -5.892 | 18/132 | AKT1,CBLB,MTOR,INSR,MAP3K1,PDPK1,MAPK1,MAPK11,RHEB,MAP2K4,SHC1,SLC2A1,SOS2,THRB,TSC2,FLOT1,MAP3K2,PRKAG2 |
| KEGG Pathway | Renal cell carcinoma | -7.5822 | -5.618 | 13/69 | AKT1,CRKL,ETS1,FH,GAB1,PAK2,PAK3,MAPK1,PTPN11,SLC2A1,SOS2,TGFA,CUL2 |
| Canonical Pathways | PID P38 ALPHA BETA DOWNSTREAM PATHWAY | -7.43748 | -5.494 | 10/38 | CREB1,ATF2,KRT19,MEF2A,MEF2C,NOS2,MAPK11,RAB5A,MAPKAPK5,HBP1 |
| Reactome Gene Sets | RAC1 GTPase cycle | -6.97192 | -5.097 | 20/185 | ABL2,JAG1,ARHGDIA,CHN2,CYBB,DOCK2,NCF4,PAK2,PAK3,PLD1,PKN2,SOS2,WAS,WIPF1,ARHGEF10,GIT2,GNA13,FERMT2,DOCK6,DOCK5 |
| KEGG Pathway | Insulin signaling pathway | -6.88553 | -5.025 | 17/137 | ACACB,AKT1,CBLB,CRKL,MTOR,INSR,PDPK1,PRKCI,MAPK1,RHEB,SHC1,SOS2,TSC2,SOCS2,FLOT1,PRKAG2,G6PC3 |
| Canonical Pathways | PID LYMPH ANGIOGENESIS PATHWAY | -6.7919 | -4.942 | 8/25 | AKT1,COL1A1,CREB1,ITGA2,MAPK1,MAPK11,MAP2K4,SHC1 |
| KEGG Pathway | Relaxin signaling pathway | -6.51535 | -4.704 | 16/129 | AKT1,ARRB1,COL1A1,COL3A1,COL4A6,CREB1,ATF2,NOS2,PRKCA,MAPK1,MAPK11,MAP2K4,SHC1,SOS2,TGFBR2,PLCB1 |
| Reactome Gene Sets | VEGFA-VEGFR2 Pathway | -6.48469 | -4.684 | 14/99 | AKT1,CDH5,CYBB,MTOR,ITGB3,NCF4,PAK2,PAK3,PDPK1,PRKCA,MAPK11,SH2D2A,ELMO1,ELMO2 |
| Reactome Gene Sets | Signaling by NTRKs | -6.28768 | -4.514 | 16/134 | BAX,CREB1,ATF2,CRKL,GAB1,ID1,JUND,MEF2A,MEF2C,NAB1,NTRK3,MAPK1,MAPK11,PTPN11,SHC1,SRF |
| KEGG Pathway | Growth hormone synthesis, secretion and action | -6.23911 | -4.472 | 15/119 | AKT1,CREB1,ATF2,CRKL,MTOR,MAP3K1,PRKCA,MAPK1,MAPK11,MAP2K4,SHC1,SOS2,STAT5B,SOCS2,PLCB1 |
| Reactome Gene Sets | Signaling by VEGF | -6.01386 | -4.277 | 14/108 | AKT1,CDH5,CYBB,MTOR,ITGB3,NCF4,PAK2,PAK3,PDPK1,PRKCA,MAPK11,SH2D2A,ELMO1,ELMO2 |
| Reactome Gene Sets | RHO GTPase Effectors | -5.64211 | -3.943 | 25/327 | AR,CDH1,CYBB,DVL3,SFN,NCF4,NUP98,PAK2,PAK3,PDPK1,PPP2R5A,PRKCA,PKN2,MAPK1,MAPK11,RAC2,RANGAP1,SRF,WAS,WIPF1,YWHAE,YWHAH,BUB3,NSL1,NCKIPSD |
| WikiPathways | Hypothesized pathways in pathogenesis of cardiovascular disease | -5.56789 | -3.881 | 7/25 | ENG,FBN1,SMAD3,SMAD4,MAPK1,SHC1,TGFBR2 |
| Canonical Pathways | PID ALK1 PATHWAY | -5.44164 | -3.774 | 7/26 | ENG,ID1,SMAD1,SMAD4,SMAD5,MAPK1,TGFBR2 |
| Reactome Gene Sets | Nuclear Events (kinase and transcription factor activation) | -5.39411 | -3.735 | 10/61 | CREB1,ATF2,ID1,JUND,MEF2A,MEF2C,NAB1,MAPK1,MAPK11,SRF |
| WikiPathways | BMP signaling in eyelid development | -5.03567 | -3.439 | 6/20 | SMAD1,SMAD4,SMAD5,MAP3K1,SFRP1,TGFA |
| Reactome Gene Sets | Toll Like Receptor 4 (TLR4) Cascade | -4.8614 | -3.299 | 14/135 | CASP8,CREB1,ATF2,ITGAM,ITGB2,MEF2A,MEF2C,MAP3K1,MAPK1,MAPK11,PTPN11,MAP2K4,RIPK2,LY86 |
| Reactome Gene Sets | Toll-like Receptor Cascades | -4.73417 | -3.197 | 15/157 | CASP8,CREB1,ATF2,CTSS,ITGAM,ITGB2,MEF2A,MEF2C,MAP3K1,MAPK1,MAPK11,PTPN11,MAP2K4,RIPK2,LY86 |
| WikiPathways | MET in type 1 papillary renal cell carcinoma | -4.64997 | -3.127 | 9/59 | AKT1,CRKL,ETS1,GAB1,PAK2,PAK3,MAPK1,PTPN11,SOS2 |
| Canonical Pathways | PID ATF2 PATHWAY | -4.64997 | -3.127 | 9/59 | CREB1,ATF2,HES1,JUND,NF1,NOS2,PRKCA,MAPK1,MAPK11 |
| KEGG Pathway | TGF-beta signaling pathway | -4.43058 | -2.953 | 11/94 | FBN1,ID1,SMAD1,SMAD3,SMAD4,SMAD5,MAPK1,TFDP1,TGFBR2,THBS1,BMP8A |
| WikiPathways | Neovascularisation processes | -4.35644 | -2.896 | 7/37 | JAG1,AKT1,SMAD1,SMAD3,SMAD5,MAPK1,CXCR4 |
| Reactome Gene Sets | Signaling by NTRK1 (TRKA) | -4.27904 | -2.834 | 12/115 | CREB1,ATF2,CRKL,ID1,JUND,MEF2A,MEF2C,NAB1,MAPK1,MAPK11,SHC1,SRF |
| WikiPathways | Osteoblast differentiation and related diseases | -4.09792 | -2.687 | 12/120 | JAG1,FGFR1,SMAD1,SMAD4,SMAD5,PRKCA,PRKCE,PRKCH,PRKCI,PRKDC,MAPK1,MAPK11 |
| Reactome Gene Sets | Interleukin-17 signaling | -3.95116 | -2.570 | 9/72 | CREB1,ATF2,MEF2A,MEF2C,MAPK1,MAPK11,MAP2K4,RIPK2,IL17RC |
| Reactome Gene Sets | MAPK targets/ Nuclear events mediated by MAP kinases | -3.86421 | -2.500 | 6/31 | CREB1,ATF2,MEF2A,MEF2C,MAPK1,MAPK11 |
| Canonical Pathways | PID CD40 PATHWAY | -3.86421 | -2.500 | 6/31 | AKT1,CBLB,CD40,MAP3K1,MAPK11,MAP2K4 |
| KEGG Pathway | TNF signaling pathway | -3.73504 | -2.398 | 11/112 | JAG1,AKT1,CASP8,CREB1,ATF2,MAPK1,MAPK11,CCL2,MAP2K4,CFLAR,ITCH |
| WikiPathways | TGF-beta receptor signaling in skeletal dysplasias | -3.6696 | -2.349 | 8/62 | ENG,FBN1,SMAD1,SMAD3,SMAD4,SMAD5,TGFBR2,THBS1 |
| Reactome Gene Sets | MAP kinase activation | -3.57265 | -2.273 | 8/64 | CREB1,ATF2,MEF2A,MEF2C,MAPK1,MAPK11,MAP2K4,RIPK2 |
| Reactome Gene Sets | MyD88-independent TLR4 cascade | -3.54579 | -2.252 | 10/99 | CASP8,CREB1,ATF2,MEF2A,MEF2C,MAPK1,MAPK11,PTPN11,MAP2K4,RIPK2 |
| Reactome Gene Sets | TRIF(TICAM1)-mediated TLR4 signaling | -3.54579 | -2.252 | 10/99 | CASP8,CREB1,ATF2,MEF2A,MEF2C,MAPK1,MAPK11,PTPN11,MAP2K4,RIPK2 |
| Reactome Gene Sets | MyD88 cascade initiated on plasma membrane | -3.39806 | -2.132 | 9/85 | CREB1,ATF2,MEF2A,MEF2C,MAP3K1,MAPK1,MAPK11,MAP2K4,RIPK2 |
| Reactome Gene Sets | Toll Like Receptor 10 (TLR10) Cascade | -3.39806 | -2.132 | 9/85 | CREB1,ATF2,MEF2A,MEF2C,MAP3K1,MAPK1,MAPK11,MAP2K4,RIPK2 |
| Reactome Gene Sets | Toll Like Receptor 5 (TLR5) Cascade | -3.39806 | -2.132 | 9/85 | CREB1,ATF2,MEF2A,MEF2C,MAP3K1,MAPK1,MAPK11,MAP2K4,RIPK2 |
| KEGG Pathway | Signaling pathways regulating pluripotency of stem cells | -3.38091 | -2.118 | 12/143 | AKT1,DVL3,FGFR1,HOXD1,ID1,SMAD1,SMAD3,SMAD4,SMAD5,MAPK1,MAPK11,KLF4 |
| Reactome Gene Sets | NGF-stimulated transcription | -3.29528 | -2.048 | 6/39 | CREB1,ATF2,ID1,JUND,NAB1,SRF |
| Reactome Gene Sets | TRAF6 mediated induction of NFkB and MAP kinases upon TLR7/8 or 9 activation | -3.17901 | -1.957 | 9/91 | CREB1,ATF2,MEF2A,MEF2C,MAP3K1,MAPK1,MAPK11,MAP2K4,RIPK2 |
| Reactome Gene Sets | MyD88 dependent cascade initiated on endosome | -3.14438 | -1.928 | 9/92 | CREB1,ATF2,MEF2A,MEF2C,MAP3K1,MAPK1,MAPK11,MAP2K4,RIPK2 |
| Reactome Gene Sets | Toll Like Receptor 7/8 (TLR7/8) Cascade | -3.11025 | -1.902 | 9/93 | CREB1,ATF2,MEF2A,MEF2C,MAP3K1,MAPK1,MAPK11,MAP2K4,RIPK2 |
| WikiPathways | TGF-beta receptor signaling | -3.09841 | -1.893 | 7/58 | ENG,SMAD1,SMAD3,SMAD4,SMAD5,TGFBR2,THBS1 |
| Reactome Gene Sets | Toll Like Receptor 3 (TLR3) Cascade | -3.07661 | -1.874 | 9/94 | CASP8,CREB1,ATF2,MEF2A,MEF2C,MAPK1,MAPK11,MAP2K4,RIPK2 |
| Reactome Gene Sets | Toll Like Receptor 9 (TLR9) Cascade | -3.01077 | -1.822 | 9/96 | CREB1,ATF2,MEF2A,MEF2C,MAP3K1,MAPK1,MAPK11,MAP2K4,RIPK2 |
| WikiPathways | Embryonic stem cell pluripotency pathways | -2.91475 | -1.743 | 10/119 | AKT1,DVL3,FGFR1,MTOR,GAB1,SMAD1,SMAD4,SMAD5,MAPK1,PTPN11 |
| Reactome Gene Sets | MyD88:MAL(TIRAP) cascade initiated on plasma membrane | -2.85403 | -1.700 | 9/101 | CREB1,ATF2,MEF2A,MEF2C,MAP3K1,MAPK1,MAPK11,MAP2K4,RIPK2 |
| Reactome Gene Sets | Toll Like Receptor TLR6:TLR2 Cascade | -2.85403 | -1.700 | 9/101 | CREB1,ATF2,MEF2A,MEF2C,MAP3K1,MAPK1,MAPK11,MAP2K4,RIPK2 |
| Reactome Gene Sets | Toll Like Receptor 2 (TLR2) Cascade | -2.76502 | -1.626 | 9/104 | CREB1,ATF2,MEF2A,MEF2C,MAP3K1,MAPK1,MAPK11,MAP2K4,RIPK2 |
| Reactome Gene Sets | Toll Like Receptor TLR1:TLR2 Cascade | -2.76502 | -1.626 | 9/104 | CREB1,ATF2,MEF2A,MEF2C,MAP3K1,MAPK1,MAPK11,MAP2K4,RIPK2 |
| Canonical Pathways | SIG CD40PATHWAYMAP | -2.73491 | -1.603 | 5/34 | CD40,MAPK1,MAPK11,MAP2K4,MAPKAPK5 |
| Reactome Gene Sets | Activation of the AP-1 family of transcription factors | -2.72194 | -1.595 | 3/10 | ATF2,MAPK1,MAPK11 |
| WikiPathways | Interleukin-1 (IL-1) structural pathway | -2.71385 | -1.588 | 6/50 | ATF2,MAP3K1,MAPK1,MAPK11,SAFB,MAP2K4 |
| Reactome Gene Sets | ERK/MAPK targets | -2.62242 | -1.514 | 4/22 | MEF2A,MEF2C,MAPK1,MAPK11 |
| WikiPathways | Host-pathogen interaction of human coronaviruses - MAPK signaling | -2.62179 | -1.514 | 5/36 | ATF2,MAP3K1,MAPK1,MAPK11,MAP2K4 |
| Canonical Pathways | PID ALK2 PATHWAY | -2.59218 | -1.490 | 3/11 | SMAD1,SMAD4,SMAD5 |
| Reactome Gene Sets | CDC42 GTPase cycle | -2.55996 | -1.465 | 11/155 | ARHGDIA,PAK2,PAK3,PLD1,ITSN1,WAS,WIPF1,ARHGEF10,GIT2,GNA13,DOCK6 |
| Canonical Pathways | SA B CELL RECEPTOR COMPLEXES | -2.47834 | -1.405 | 4/24 | ATF2,MAP3K1,MAPK1,SHC1 |
| KEGG Pathway | Fluid shear stress and atherosclerosis | -2.41742 | -1.356 | 10/139 | AKT1,CDH5,GSTM4,ITGB3,MEF2A,MEF2C,MAPK11,RAC2,CCL2,MAP2K4 |
| WikiPathways | BMP2-WNT4-FOXO1 pathway in primary endometrial stromal cell differentiation | -2.37035 | -1.322 | 3/13 | SMAD1,SMAD5,SFRP1 |

**Table 3. Comparison of DEGs between db/db *vs.* db/m control and combinatory treatment *vs.* db/db group.** There are 181 genes which were significantly upregulated in DKD model (db/db mice) but were downregulated in anti-VEGF-B and anti-IL-17A combined treatment group. These genes were labeled as “Down reversed” genes to indicate the outcome of combinatory treatment from the transcriptomics perspective.

| **Gene Name** | **Log2FC (db/db *vs.* db/m)** | **adjP (db/db *vs.* db/m)** | **Log2FC (Treatment *vs.* db/db)** | **adjP (Treatment *vs.* db/db)** | **Outcome of Treatment** |
| --- | --- | --- | --- | --- | --- |
| Slc22a7 | -7.70602323 | 2.20E-48 | 3.722738 | 4.63E-06 | Up reversed |
| Cyp7b1 | -5.077561915 | 1.92E-11 | 2.103728 | 0.017155048 | Up reversed |
| Epo | -4.42374122 | 8.95E-05 | 4.663272 | 0.000110781 | Up reversed |
| Apoh | -3.653442558 | 3.38E-05 | 2.958261 | 0.007075433 | Up reversed |
| Hmox1 | -3.160820206 | 5.71E-05 | 2.170063 | 5.88E-05 | Up reversed |
| Cyp2a4 | -2.992482687 | 0.000174478 | 1.929645 | 0.01538888 | Up reversed |
| Lars2 | -2.906943898 | 0.037117769 | 2.121292 | 0.04537064 | Up reversed |
| Upp2 | -2.828166766 | 0.002290117 | 2.810085 | 0.001679169 | Up reversed |
| Plau | -2.516908256 | 0.000719191 | 1.654828 | 0.025684626 | Up reversed |
| C8a | -2.478378546 | 0.012038951 | 2.016166 | 0.041479421 | Up reversed |
| Stk32c | -2.405060477 | 0.001004581 | 2.1694 | 0.001126488 | Up reversed |
| Aadat | -2.219718982 | 0.01108115 | 1.78785 | 0.03613205 | Up reversed |
| Tgtp2 | -2.098894935 | 0.004963191 | 2.162895 | 0.002675176 | Up reversed |
| Tgtp1 | -2.037614255 | 0.008235651 | 2.043421 | 0.004881917 | Up reversed |
| Nuak2 | -1.679557908 | 0.024141036 | 1.617671 | 0.008360832 | Up reversed |
| Bcl6 | 1.568736199 | 0.037117769 | -1.66466 | 0.019509585 | Down reversed |
| Ormdl3 | 1.634192711 | 0.031479931 | -1.64608 | 0.020334722 | Down reversed |
| Snapc1 | 1.683894946 | 0.044902478 | -1.72389 | 0.026645017 | Down reversed |
| Col4a5 | 1.704577568 | 0.025088906 | -1.77994 | 0.017746336 | Down reversed |
| Sgpl1 | 1.741504834 | 0.030601655 | -1.72396 | 0.022974437 | Down reversed |
| Pcyox1 | 1.743151767 | 0.022465097 | -1.88941 | 0.007215886 | Down reversed |
| Mbp | 1.761842227 | 0.026204055 | -1.59025 | 0.035482295 | Down reversed |
| Fam171a2 | 1.769781035 | 0.043763862 | -1.64897 | 0.03984328 | Down reversed |
| Gm21451 | 1.779815384 | 0.027944293 | -1.68616 | 0.023404811 | Down reversed |
| Tmtc1 | 1.78354849 | 0.040669841 | -2.08611 | 0.005230824 | Down reversed |
| Elovl5 | 1.789687215 | 0.03764918 | -2.30318 | 0.002013982 | Down reversed |
| Slc45a3 | 1.827282663 | 0.024300439 | -1.76449 | 0.013038684 | Down reversed |
| Sgk1 | 1.827673732 | 0.037322872 | -2.13664 | 0.002675176 | Down reversed |
| Stom | 1.83979587 | 0.01569782 | -1.84985 | 0.013582257 | Down reversed |
| Btbd3 | 1.869801108 | 0.028889111 | -1.70279 | 0.043996573 | Down reversed |
| Esrp1 | 1.880366792 | 0.043763862 | -1.86841 | 0.028329175 | Down reversed |
| Fam49b | 1.914606045 | 0.03551392 | -1.78737 | 0.041520374 | Down reversed |
| Mras | 1.914798132 | 0.027282995 | -2.12338 | 0.007215886 | Down reversed |
| Stard13 | 1.922603691 | 0.014898745 | -1.71176 | 0.024438947 | Down reversed |
| Ncam1 | 1.925425243 | 0.008468582 | -1.74493 | 0.0169021 | Down reversed |
| Alcam | 1.935839836 | 0.02270024 | -2.1271 | 0.005793179 | Down reversed |
| Pik3c2b | 1.937158751 | 0.036252911 | -1.91679 | 0.023404811 | Down reversed |
| Satb1 | 1.949030143 | 0.043241747 | -1.8138 | 0.03953099 | Down reversed |
| Slc7a6 | 1.958748174 | 0.02223175 | -1.67622 | 0.041044902 | Down reversed |
| Mr1 | 1.962775232 | 0.022115557 | -1.65865 | 0.032398031 | Down reversed |
| Tbx3 | 1.980460543 | 0.04025064 | -2.26477 | 0.008360832 | Down reversed |
| Galm | 1.981508297 | 0.0345078 | -2.5585 | 0.001579685 | Down reversed |
| Ambp | 2.024759902 | 0.02223175 | -1.60153 | 0.032032273 | Down reversed |
| Nrgn | 2.04532106 | 0.03262653 | -2.04453 | 0.02038859 | Down reversed |
| Cpne2 | 2.047721853 | 0.044873111 | -2.22228 | 0.018750739 | Down reversed |
| Aox1 | 2.079590633 | 0.035998131 | -2.27201 | 0.012637066 | Down reversed |
| Dixdc1 | 2.093585432 | 0.003442949 | -1.8592 | 0.008360832 | Down reversed |
| Igfbp6 | 2.094903782 | 0.030838291 | -2.55113 | 0.007215886 | Down reversed |
| Tmem45b | 2.101558906 | 0.026772059 | -2.82389 | 0.001413202 | Down reversed |
| Crem | 2.113802636 | 0.037117769 | -2.22068 | 0.017000053 | Down reversed |
| Lix1 | 2.115081462 | 0.022388325 | -2.41145 | 0.004830527 | Down reversed |
| Adam10 | 2.11616473 | 0.016794364 | -2.39695 | 0.003921349 | Down reversed |
| Mpzl2 | 2.116642198 | 0.016281618 | -1.92332 | 0.016931817 | Down reversed |
| Hebp2 | 2.125031662 | 0.049318932 | -2.2863 | 0.020334722 | Down reversed |
| Rab15 | 2.126701096 | 0.028889111 | -2.22281 | 0.014117217 | Down reversed |
| Ptgs1 | 2.149412764 | 0.006914965 | -2.15953 | 0.004070284 | Down reversed |
| Hif1a | 2.150439984 | 0.023824817 | -2.32117 | 0.007215886 | Down reversed |
| Casp3 | 2.150720354 | 0.01476865 | -2.14802 | 0.009692415 | Down reversed |
| Mier3 | 2.153214315 | 0.013452397 | -2.20825 | 0.006919189 | Down reversed |
| Plk4 | 2.172177509 | 0.030667142 | -2.10225 | 0.032406969 | Down reversed |
| Plat | 2.174043011 | 0.047340206 | -2.63778 | 0.008801054 | Down reversed |
| Cdon | 2.197951448 | 0.018073312 | -2.06907 | 0.021191009 | Down reversed |
| Slc2a8 | 2.201855002 | 0.001213592 | -1.86084 | 0.006590289 | Down reversed |
| Fgfr2 | 2.253160406 | 0.005149846 | -2.48362 | 0.001421344 | Down reversed |
| Srpx2 | 2.299893861 | 0.01873698 | -1.9424 | 0.02499142 | Down reversed |
| Reep2 | 2.307524175 | 0.016099841 | -2.03748 | 0.028103751 | Down reversed |
| Lhfpl2 | 2.307624656 | 0.011289535 | -2.36813 | 0.006467074 | Down reversed |
| Agmo | 2.3153673 | 0.003542788 | -2.26436 | 0.001802178 | Down reversed |
| S100a6 | 2.334617516 | 0.01873698 | -2.5144 | 0.003709526 | Down reversed |
| Ppp1r15a | 2.335358457 | 0.016794364 | -2.04524 | 0.034088648 | Down reversed |
| Krt18 | 2.33834997 | 0.033522435 | -2.55546 | 0.006467074 | Down reversed |
| Rhou | 2.354824989 | 0.041795029 | -2.33254 | 0.024038582 | Down reversed |
| Hspb8 | 2.372446826 | 0.001103053 | -1.84431 | 0.012195731 | Down reversed |
| Endod1 | 2.376686043 | 0.041285625 | -3.33031 | 0.000499096 | Down reversed |
| B3gnt7 | 2.420230416 | 0.022445341 | -2.94356 | 0.002021091 | Down reversed |
| Plch2 | 2.444749392 | 0.047148327 | -2.33734 | 0.043659804 | Down reversed |
| Shh | 2.46025712 | 0.017184246 | -2.90237 | 0.002162867 | Down reversed |
| Sesn3 | 2.463973381 | 0.022115557 | -2.83635 | 0.003198292 | Down reversed |
| Lgals3bp | 2.491132449 | 0.002180986 | -2.30992 | 0.004375311 | Down reversed |
| Btg2 | 2.49200905 | 0.013117415 | -1.95019 | 0.046444197 | Down reversed |
| Nbl1 | 2.499481577 | 0.021768597 | -2.78976 | 0.002693736 | Down reversed |
| Arhgap8 | 2.534603963 | 0.015894607 | -2.47057 | 0.009451409 | Down reversed |
| Upk1a | 2.536878101 | 0.026588178 | -3.1114 | 0.003198292 | Down reversed |
| Ppp1r3c | 2.554706425 | 0.011319059 | -2.77171 | 0.003921349 | Down reversed |
| Tspan6 | 2.569975154 | 0.035387443 | -3.57731 | 0.000772887 | Down reversed |
| Elmod1 | 2.573795269 | 0.020675424 | -2.78911 | 0.007215886 | Down reversed |
| Arhgap28 | 2.582904335 | 0.000490795 | -1.67498 | 0.028242817 | Down reversed |
| Tnfrsf11a | 2.589976979 | 0.023481875 | -2.72479 | 0.007301271 | Down reversed |
| Frmpd4 | 2.624018818 | 0.036298537 | -3.4488 | 0.003899994 | Down reversed |
| Car8 | 2.63022021 | 0.00148337 | -2.22312 | 0.007215886 | Down reversed |
| Fgf12 | 2.639191637 | 0.015997127 | -2.38964 | 0.020544325 | Down reversed |
| Mgat4a | 2.648816608 | 0.009852434 | -2.74032 | 0.002917929 | Down reversed |
| Ociad2 | 2.649575219 | 0.00918649 | -3.07319 | 0.001802178 | Down reversed |
| Hey1 | 2.66025507 | 0.003749668 | -2.64534 | 0.003957724 | Down reversed |
| Mpzl1 | 2.695067107 | 0.001554938 | -2.42353 | 0.003921349 | Down reversed |
| Akap12 | 2.706974106 | 0.026795367 | -2.84108 | 0.008801054 | Down reversed |
| Itm2c | 2.71832434 | 0.014898745 | -3.37972 | 0.000789913 | Down reversed |
| Ros1 | 2.722574088 | 0.040672685 | -3.53767 | 0.004028843 | Down reversed |
| Nav1 | 2.725964377 | 0.00148337 | -2.30879 | 0.008001147 | Down reversed |
| Hspa2 | 2.752450414 | 0.004742836 | -2.87117 | 0.002245781 | Down reversed |
| Stxbp1 | 2.79546242 | 0.005527511 | -2.61319 | 0.006775235 | Down reversed |
| Cpxm2 | 2.816472033 | 0.009002429 | -3.03543 | 0.00704618 | Down reversed |
| Vwf | 2.846018815 | 0.000633235 | -3.17354 | 7.04E-05 | Down reversed |
| Pbx4 | 2.846674547 | 0.00014563 | -1.80122 | 0.020072331 | Down reversed |
| Lamb3 | 2.851841778 | 0.018896187 | -4.01749 | 7.41E-05 | Down reversed |
| Heg1 | 2.887523082 | 0.003314092 | -2.88355 | 0.002609643 | Down reversed |
| Camk1d | 2.895091823 | 0.023481875 | -3.04696 | 0.010483033 | Down reversed |
| Sdk1 | 2.91891993 | 0.004402658 | -2.67601 | 0.009342129 | Down reversed |
| Mtus2 | 2.937317348 | 0.005411999 | -4.6949 | 8.23E-06 | Down reversed |
| Hspa1a | 2.946913094 | 0.022115557 | -3.40952 | 0.004180493 | Down reversed |
| Klf5 | 2.986121977 | 0.028889111 | -3.29666 | 0.008360832 | Down reversed |
| Col4a6 | 2.999859159 | 0.017537831 | -3.77841 | 0.000678346 | Down reversed |
| Cntn1 | 3.001593227 | 0.013221753 | -2.98633 | 0.009302908 | Down reversed |
| Tmod2 | 3.004518834 | 0.009002429 | -3.56906 | 0.001413202 | Down reversed |
| Fam129a | 3.006755562 | 0.003858175 | -3.17453 | 0.001679169 | Down reversed |
| Nyap1 | 3.01383186 | 0.000777334 | -2.84382 | 0.001802178 | Down reversed |
| Wnt4 | 3.029014341 | 0.022115557 | -4.07659 | 0.001320405 | Down reversed |
| Igfbp2 | 3.03060774 | 0.001423357 | -4.80875 | 8.23E-06 | Down reversed |
| Nfe2l3 | 3.037405125 | 0.015641842 | -4.39026 | 7.55E-05 | Down reversed |
| Dab1 | 3.041789921 | 0.000554157 | -2.15471 | 0.017746336 | Down reversed |
| Upk2 | 3.066017022 | 0.049072359 | -5.02121 | 0.000678346 | Down reversed |
| Rtn1 | 3.071919647 | 0.044873111 | -4.40481 | 0.000446468 | Down reversed |
| Fbxo2 | 3.078153394 | 0.013117415 | -2.77996 | 0.012431726 | Down reversed |
| Oaz3 | 3.080741509 | 0.025088906 | -2.77585 | 0.029503818 | Down reversed |
| Kcnj13 | 3.091704716 | 0.020675424 | -2.9346 | 0.021418757 | Down reversed |
| Homer2 | 3.091713285 | 0.028642868 | -4.36667 | 0.001720773 | Down reversed |
| Pkhd1l1 | 3.121887852 | 0.003665894 | -3.53265 | 0.000827107 | Down reversed |
| Prkar2b | 3.138789719 | 0.020626108 | -5.3502 | 1.10E-05 | Down reversed |
| Dlgap1 | 3.185254034 | 0.029441011 | -4.4979 | 0.00191194 | Down reversed |
| Gpc2 | 3.187332075 | 0.003442949 | -2.42478 | 0.025233545 | Down reversed |
| Hspa1b | 3.204146317 | 0.033832176 | -4.12909 | 0.002068724 | Down reversed |
| Foxa1 | 3.23442607 | 0.022127264 | -4.25991 | 0.002675176 | Down reversed |
| Gucy2g | 3.242503748 | 0.0459697 | -5.38642 | 0.000462485 | Down reversed |
| Spink8 | 3.245212456 | 0.000901845 | -3.0624 | 0.001795224 | Down reversed |
| Rnf182 | 3.260427772 | 0.035894099 | -4.05293 | 0.007215886 | Down reversed |
| Tmprss4 | 3.262177178 | 0.000777736 | -1.99214 | 0.025995869 | Down reversed |
| Pde1c | 3.26375183 | 0.001629913 | -3.6453 | 0.000601147 | Down reversed |
| Pdgfrl | 3.322889308 | 0.019957998 | -3.32718 | 0.0169021 | Down reversed |
| Slc8a3 | 3.432415576 | 0.013686323 | -5.1696 | 0.000167417 | Down reversed |
| Ahrr | 3.473462467 | 0.013452397 | -4.16769 | 0.000576389 | Down reversed |
| Gm15753 | 3.499058987 | 0.007396543 | -3.68842 | 0.004669544 | Down reversed |
| Fer1l4 | 3.516240327 | 0.018896187 | -5.01376 | 0.000330855 | Down reversed |
| Actg2 | 3.530018251 | 0.032423996 | -6.37594 | 5.83E-05 | Down reversed |
| Celf3 | 3.56697964 | 0.010724569 | -4.36721 | 0.001006468 | Down reversed |
| Muc13 | 3.57444577 | 0.029982995 | -3.56749 | 0.008801054 | Down reversed |
| Cthrc1 | 3.642851795 | 0.015894607 | -5.55767 | 0.00035724 | Down reversed |
| Sprr1a | 3.672910183 | 0.014025767 | -3.40654 | 0.010272924 | Down reversed |
| Krt88 | 3.700617669 | 0.020675424 | -3.82762 | 0.010882623 | Down reversed |
| Gper1 | 3.720667434 | 0.000373434 | -3.10866 | 0.003250949 | Down reversed |
| Lgi3 | 3.724083692 | 0.000490795 | -3.53539 | 0.001147343 | Down reversed |
| Nr4a2 | 3.729128649 | 0.00095425 | -3.64419 | 0.001413202 | Down reversed |
| Megf9 | 3.736443898 | 0.001810973 | -3.35022 | 0.004027449 | Down reversed |
| Krt87 | 3.763477102 | 0.011289535 | -3.54877 | 0.011383165 | Down reversed |
| Gem | 3.76411074 | 0.004638758 | -4.13148 | 0.000787572 | Down reversed |
| Cldn11 | 3.776246453 | 0.032235649 | -7.5765 | 6.69E-07 | Down reversed |
| Tbx18 | 3.818336205 | 0.049158535 | -4.80116 | 0.015508883 | Down reversed |
| Kif5c | 3.825737617 | 0.000516585 | -2.71151 | 0.023082243 | Down reversed |
| Gcnt3 | 3.826935222 | 0.03262653 | -6.83641 | 8.79E-06 | Down reversed |
| Upk1b | 3.873033552 | 0.0062699 | -4.45772 | 0.001637751 | Down reversed |
| Grem2 | 3.900351532 | 0.005871293 | -3.54338 | 0.002162867 | Down reversed |
| Tnfsf9 | 4.001285511 | 0.004638758 | -3.93275 | 0.004027449 | Down reversed |
| Bmp3 | 4.049726805 | 0.001607529 | -3.52729 | 0.002775841 | Down reversed |
| Crabp2 | 4.053149595 | 0.003442949 | -5.76065 | 2.88E-05 | Down reversed |
| Rspo3 | 4.055219269 | 0.006627738 | -6.22109 | 3.47E-05 | Down reversed |
| Crybb1 | 4.128050695 | 0.007585044 | -4.82127 | 0.002609643 | Down reversed |
| Ctse | 4.181418644 | 0.005075967 | -4.14712 | 0.004306587 | Down reversed |
| Ak8 | 4.266695487 | 0.016794364 | -3.6458 | 0.020334722 | Down reversed |
| Slc10a4l | 4.306172415 | 0.001429952 | -5.13775 | 0.000279149 | Down reversed |
| Gsdmc4 | 4.391462638 | 0.014898745 | -4.75638 | 0.001413202 | Down reversed |
| Tbx3os2 | 4.429374433 | 0.004377852 | -3.92433 | 0.008255797 | Down reversed |
| Adgra1 | 4.431286389 | 0.00117636 | -3.5153 | 0.004638827 | Down reversed |
| Crisp3 | 4.478178683 | 0.029441011 | -7.34429 | 0.002160744 | Down reversed |
| Muc4 | 4.510815349 | 0.017537831 | -4.15298 | 0.012101018 | Down reversed |
| Vtcn1 | 4.577939248 | 4.66E-05 | -3.64654 | 0.000955603 | Down reversed |
| Atp2c2 | 4.583125863 | 0.000233761 | -3.61125 | 0.003370907 | Down reversed |
| Akr1b7 | 4.585187101 | 0.000191235 | -3.57276 | 0.004638827 | Down reversed |
| Slc14a2 | 4.636189716 | 0.020675424 | -4.26389 | 0.020782377 | Down reversed |
| Dlx3 | 4.637921001 | 0.027282995 | -4.15776 | 0.021698622 | Down reversed |
| Tnnc2 | 4.643528488 | 0.004742836 | -3.58821 | 0.0169021 | Down reversed |
| Mir31 | 4.704469454 | 0.000777334 | -4.15733 | 0.001871092 | Down reversed |
| Tnf | 4.72179792 | 0.000868145 | -3.1096 | 0.029749332 | Down reversed |
| Hk2 | 4.745914499 | 0.0002105 | -3.8369 | 0.003008744 | Down reversed |
| Alox12e | 4.795773598 | 0.009348472 | -4.29537 | 0.01364316 | Down reversed |
| Zan | 4.823767336 | 0.000719191 | -3.58877 | 0.005964451 | Down reversed |
| Shisal1 | 4.899595091 | 1.66E-05 | -4.29619 | 7.75E-06 | Down reversed |
| Fut2 | 5.131246171 | 0.000910433 | -4.14704 | 0.0064223 | Down reversed |
| Obscn | 5.50260286 | 0.000206322 | -4.6474 | 0.002791897 | Down reversed |
| Gm4265 | 5.619637694 | 0.008056482 | -7.67633 | 0.00171817 | Down reversed |
| 2310069B03Rik | 5.716687817 | 0.005689565 | -5.88237 | 0.00336489 | Down reversed |
| Itga2 | 5.964179557 | 0.001213592 | -7.70437 | 2.03E-05 | Down reversed |
| Ano2 | 6.094330341 | 8.61E-05 | -7.54764 | 1.10E-05 | Down reversed |
| Tm4sf20 | 6.151635279 | 0.00049734 | -5.76336 | 0.000601717 | Down reversed |
| Prss35 | 6.996149449 | 0.000710676 | -12.6204 | 7.75E-06 | Down reversed |
| Gm20556 | 7.29601224 | 0.002377845 | -4.60847 | 0.017506745 | Down reversed |
| Zfp872 | 7.404584401 | 0.00079665 | -7.47128 | 0.000787572 | Down reversed |
| Fgf15 | 9.651651963 | 0.000105982 | -9.71938 | 0.000117542 | Down reversed |

**Table 4. Metascape pathway enrichment analysis of 181 down reversed genes.**

| **Enrichment Data Source** | **Pathway Description** | **LogP** | **Log(q-value)** | **Enriched Gene Ratio** | **Enriched Genes** |
| --- | --- | --- | --- | --- | --- |
| Canonical Pathways | NABA CORE MATRISOME | -5.32062 | -2.321 | 10/275 | COL4A5,COL4A6,IGFBP2,IGFBP6,LAMB3,VWF,SRPX2,RSPO3,CTHRC1,LGI3 |
| Canonical Pathways | NABA MATRISOME ASSOCIATED | -5.1092 | -2.211 | 16/751 | ADAM10,AMBP,BMP3,CTSE,MEGF9,FGF12,MUC4,PLAT,S100A6,SHH,TNF,TNFSF9,FGF19,WNT4,MUC13,GPC2 |
| WikiPathways | Focal adhesion: PI3K-Akt-mTOR-signaling pathway | -4.87904 | -2.037 | 10/309 | COL4A6,FGF12,FGFR2,HIF1A,FOXA1,ITGA2,LAMB3,PIK3C2B,VWF,FGF19 |
| Canonical Pathways | NABA ECM GLYCOPROTEINS | -4.71946 | -1.903 | 8/196 | IGFBP2,IGFBP6,LAMB3,VWF,SRPX2,RSPO3,CTHRC1,LGI3 |
| Reactome Gene Sets | Laminin interactions | -4.5882 | -1.818 | 4/30 | COL4A5,COL4A6,ITGA2,LAMB3 |
| WikiPathways | Malignant pleural mesothelioma | -4.37818 | -1.648 | 11/429 | ACTG2,COL4A5,COL4A6,FGF12,FGFR2,HIF1A,ITGA2,LAMB3,FGF19,WNT4,CTHRC1 |
| KEGG Pathway | Toxoplasmosis | -4.29643 | -1.608 | 6/113 | ALOX5,CASP3,HSPA1A,HSPA2,LAMB3,TNF |
| WikiPathways | Spinal cord injury | -4.15073 | -1.522 | 6/120 | CASP3,GFAP,MBP,ROS1,TNF,BTG2 |
| Reactome Gene Sets | Anchoring fibril formation | -4.09177 | -1.494 | 3/15 | COL4A5,COL4A6,LAMB3 |
| KEGG Pathway | ECM-receptor interaction | -3.94488 | -1.393 | 5/82 | COL4A5,COL4A6,ITGA2,LAMB3,VWF |
| KEGG Pathway | MAPK signaling pathway | -3.91482 | -1.393 | 8/255 | CASP3,FGF12,FGFR2,HSPA1A,HSPA2,TNF,FGF19,MRAS |
| KEGG Pathway | Pathways in cancer | -3.86712 | -1.357 | 12/576 | CASP3,COL4A5,COL4A6,FGF12,FGFR2,HIF1A,ITGA2,LAMB3,SHH,FGF19,HEY1,WNT4 |
| Reactome Gene Sets | Muscle contraction | -3.81896 | -1.349 | 7/197 | ACTG2,FGF12,NPR2,SLC8A3,TNNC2,DYSF,TMOD2 |
| KEGG Pathway | PI3K-Akt signaling pathway | -3.76016 | -1.313 | 9/342 | COL4A5,COL4A6,FGF12,FGFR2,ITGA2,LAMB3,SGK1,VWF,FGF19 |
| Canonical Pathways | NABA SECRETED FACTORS | -3.75073 | -1.311 | 9/343 | BMP3,MEGF9,FGF12,S100A6,SHH,TNF,TNFSF9,FGF19,WNT4 |
| KEGG Pathway | Proteoglycans in cancer | -3.73884 | -1.311 | 7/203 | CASP3,HIF1A,ITGA2,SHH,TNF,MRAS,WNT4 |
| KEGG Pathway | Cell adhesion molecules (CAMs) | -3.7156 | -1.299 | 6/144 | ALCAM,CNTN1,NCAM1,CLDN11,MPZL1,VTCN1 |
| Canonical Pathways | PID CASPASE PATHWAY | -3.66913 | -1.272 | 4/51 | CASP3,KRT18,SATB1,TNF |
| Reactome Gene Sets | Signaling by Receptor Tyrosine Kinases | -3.63693 | -1.258 | 11/521 | ADAM10,COL4A5,FGFR2,GFAP,HIF1A,ITGA2,LAMB3,PLAT,SGK1,FGF19,ESRP1 |
| KEGG Pathway | Amoebiasis | -3.62272 | -1.249 | 5/96 | CASP3,COL4A5,COL4A6,LAMB3,TNF |
| KEGG Pathway | Small cell lung cancer | -3.5605 | -1.211 | 5/99 | CASP3,COL4A5,COL4A6,ITGA2,LAMB3 |
| KEGG Pathway | Legionellosis | -3.54184 | -1.211 | 4/55 | CASP3,HSPA1A,HSPA2,TNF |
| Reactome Gene Sets | Extracellular matrix organization | -3.42787 | -1.157 | 8/301 | ADAM10,CASP3,COL4A5,COL4A6,ITGA2,LAMB3,NCAM1,VWF |
| Reactome Gene Sets | Non-integrin membrane-ECM interactions | -3.4243 | -1.157 | 4/59 | COL4A5,COL4A6,ITGA2,LAMB3 |
| KEGG Pathway | AGE-RAGE signaling pathway in diabetic complications | -3.40442 | -1.157 | 5/107 | CASP3,CNTN1,COL4A5,COL4A6,TNF |
| WikiPathways | Epithelial to mesenchymal transition in colorectal cancer | -3.39801 | -1.154 | 6/165 | COL4A5,COL4A6,HIF1A,CLDN11,WNT4,TMPRSS4 |
| Reactome Gene Sets | Interleukin-4 and Interleukin-13 signaling | -3.38584 | -1.146 | 5/108 | ALOX5,BCL6,HIF1A,TNF,RHOU |
| WikiPathways | Burn wound healing | -3.34925 | -1.119 | 5/110 | AMBP,CASP3,FGFR2,S100A6,TNF |
| Canonical Pathways | PID INTEGRIN1 PATHWAY | -3.23824 | -1.042 | 4/66 | COL4A5,COL4A6,ITGA2,LAMB3 |
| WikiPathways | Oligodendrocyte specification and differentiation, leading to myelin components for CNS | -3.12644 | -0.993 | 3/31 | MBP,SHH,TNF |
| WikiPathways | Hair follicle development: organogenesis - part 2 of 3 | -3.04596 | -0.943 | 3/33 | FGFR2,NCAM1,SHH |
| Reactome Gene Sets | ECM proteoglycans | -3.00714 | -0.923 | 4/76 | COL4A5,COL4A6,ITGA2,NCAM1 |
| Reactome Gene Sets | Degradation of the extracellular matrix | -2.8769 | -0.850 | 5/140 | ADAM10,CASP3,COL4A5,COL4A6,LAMB3 |
| Reactome Gene Sets | Integrin cell surface interactions | -2.82637 | -0.816 | 4/85 | COL4A5,COL4A6,ITGA2,VWF |
| Canonical Pathways | NABA BASEMENT MEMBRANES | -2.80088 | -0.802 | 3/40 | COL4A5,COL4A6,LAMB3 |
| KEGG Pathway | Protein digestion and absorption | -2.73498 | -0.760 | 4/90 | COL4A5,COL4A6,KCNJ13,SLC8A3 |
| WikiPathways | Genes controlling nephrogenesis | -2.68093 | -0.737 | 3/44 | FGFR2,SHH,WNT4 |
| WikiPathways | Sudden infant death syndrome (SIDS) susceptibility pathways | -2.59951 | -0.700 | 5/162 | CASP3,CREM,HIF1A,PRKAR2B,TNF |
| Canonical Pathways | PID FGF PATHWAY | -2.40412 | -0.589 | 3/55 | FGFR2,NCAM1,FGF19 |
| Reactome Gene Sets | L1CAM interactions | -2.29834 | -0.516 | 4/119 | ALCAM,CNTN1,ITGA2,NCAM1 |
| WikiPathways | Osteoblast differentiation and related diseases | -2.28554 | -0.507 | 4/120 | FGFR2,PIK3C2B,HEY1,WNT4 |
| Reactome Gene Sets | Assembly of collagen fibrils and other multimeric structures | -2.27774 | -0.500 | 3/61 | COL4A5,COL4A6,LAMB3 |
| KEGG Pathway | Focal adhesion | -2.22121 | -0.468 | 5/199 | COL4A5,COL4A6,ITGA2,LAMB3,VWF |
| Reactome Gene Sets | Collagen degradation | -2.21961 | -0.468 | 3/64 | ADAM10,COL4A5,COL4A6 |
| KEGG Pathway | Central carbon metabolism in cancer | -2.2009 | -0.456 | 3/65 | FGFR2,HIF1A,HK2 |
| WikiPathways | Adipogenesis | -2.1524 | -0.425 | 4/131 | BMP3,KLF5,HIF1A,TNF |
| WikiPathways | Ectoderm differentiation | -2.01107 | -0.353 | 4/144 | FGFR2,CLDN11,SHH,CDON |
| KEGG Pathway | Estrogen signaling pathway | -2.00083 | -0.348 | 4/145 | GPER1,HSPA1A,HSPA2,KRT18 |
