# Supplementary figures and images for: Simultaneous blockade of VEGF-B and IL-17A ameliorated diabetic kidney disease by reducing ectopic lipid deposition and alleviating inflammation response
Source: Cell Death Discov. 2023 Jan 16;9:8. doi: 10.1038/s41420-023-01304-5 (PMC9842640; doi:10.1038/s41420-023-01304-5)

**Fig. 5D p-NF-κB 65kDa, β-actin 42kDa**

**
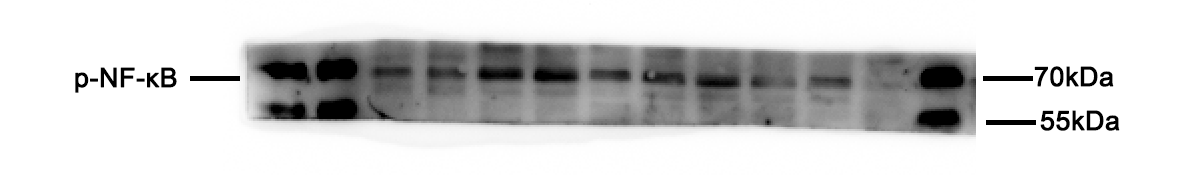
**

**
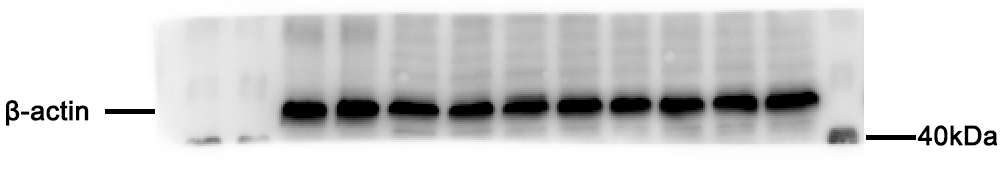
**

**Fig. S2C FATP4 72kDa, GAPDH 37kDa**

**
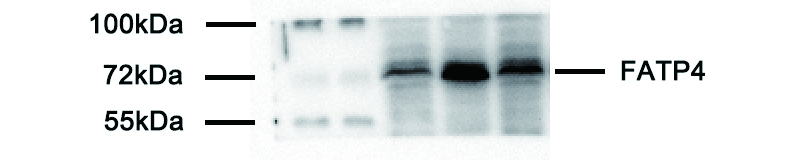
**

**
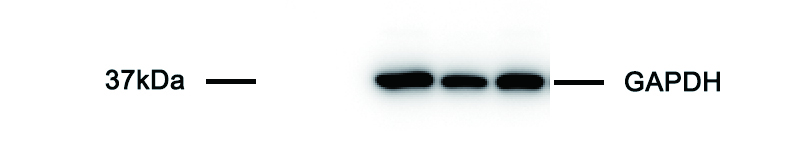
**

Supplement: Supplementary file 2 — Original Data File [file 41420_2023_1304_MOESM2_ESM.docx]
